# Adversarial Patch and Camouflage Attacks on Aerial Object Detection: A Geometry-Aware Review

**DOI:** 10.3390/s26196057

**Published:** 2026-09-24

**Authors:** Sandesh Shrestha, K.T. Yasas Mahima, Asanka G. Perera

**Affiliations:** 1 Industrial Systems Engineering (ISE), Asian Institute of Technology (AIT), Pathum Thani 12120, Thailand; sandeshshrestha45@gmail.com; 2 School of Engineering and Technology, University of New South Wales, Canberra, ACT 2612, Australia; yasas.mahima@unsw.edu.au; 3 School of Engineering & Digital Technologies, University of Southern Queensland, Springfield Central, QLD 4300, Australia

**Keywords:** adversarial patch, adversarial camouflage, object detection, uncrewed aerial vehicles, remote sensing, physical adversarial attacks

## Abstract

Uncrewed aerial vehicles increasingly rely on on-board deep object detectors for applications such as surveillance, delivery, agriculture, and traffic monitoring. The vulnerability of these detectors to adversarial patches has therefore become a practical safety concern. Research on adversarial patch and camouflage attacks against aerial object detection has expanded rapidly, yet existing reviews consider aerial systems only as one application domain among many and do not examine the unique geometric conditions of aerial imagery. This review surveys adversarial patch and camouflage attacks, together with the corresponding defense mechanisms, for aerial, UAV, and remote sensing object detection. The literature is organized using a taxonomy based on two dimensions: the physical medium of the perturbation and the application domain it targets. Existing methods are then compared with respect to physical robustness, evaluation datasets and detectors, threat models, and their treatment of viewpoint variation. A recurring observation across the literature is that viewpoint variation is represented primarily through object scale, while explicit modeling of viewing geometry remains limited. This trend is closely linked to the characteristics of the aerial datasets used for evaluation, which typically provide realistic imagery but limited geometric information. Consequently, questions concerning viewpoint-dependent placement, degradation, and naturalness remain only partially explored. The review concludes by identifying the major research gaps, future directions, and deployment challenges that are likely to shape the next generation of aerial adversarial attack and defense research.

## 1. Introduction

Uncrewed aerial vehicles (UAVs) have evolved from niche platforms into widely used tools for surveillance, package delivery, precision agriculture, infrastructure inspection, and traffic monitoring. Most of these applications employ neural network-based object detectors, running either on-board or on a downlinked video stream, to detect and localize vehicles, people, infrastructure, and other objects in aerial imagery. Some systems go further and label the scene at the pixel level, using semantic or instance segmentation to outline a target rather than enclose it in a bounding box [1]. However, the same detectors that make UAVs useful inherit a well-documented vulnerability of deep vision models, as they can be fooled by adversarial inputs such as printable adversarial patches designed to suppress or alter detections when captured within the camera’s field of view [2,3].

A patch that reliably hides a vehicle or person from a drone-based detector is a practical means of defeating aerial surveillance, and because such attacks are physically realizable, they need no access to the detector at inference time. Figure 1 shows an example of this threat using an aerial image by depicting a scene that is correctly interpreted by the detector alongside the same scene after a small adversarial patch suppresses detection. As UAVs become autonomous and are deployed in security-critical applications, these adversarial patch attacks pose an increasing threat to aerial perception systems. Evaluating the adversarial vulnerabilities of such UAV-based aerial perception systems and the robustness of available defenses is therefore essential to ensuring reliable and secure aerial perception.

Research on adversarial patch attacks has attracted increasing attention in recent years. Early works mainly focused on patch-based attacks against image classification and person detection systems [2,3]. Subsequent studies expanded to encompass overhead and oblique imagery [4,5,6], three-dimensional vehicle camouflage [7,8,9], thermal and multimodal sensing [10,11,12], pixel-level segmentation [13], and various defense mechanisms [14,15,16]. Nevertheless, aerial and UAV settings have attracted far less attention than ground-level imagery. Several review papers have examined physical adversarial attacks, adversarial patches, and object-detection attacks from broader computer vision, security, and autonomous-driving perspectives [17,18,19,20,21,22,23,24]. While these studies provide broad coverage of the field, UAV imagery is typically treated as one application domain among many, with comparatively limited attention given to the unique environmental conditions and challenges associated with aerial platforms. Furthermore, UAVs observe targets from varying altitudes and viewing angles, and these parameters can change from frame to frame, which is a vital consideration when developing patch attacks.

Hence, this review specifically focuses on adversarial attacks against aerial object detection and the unique challenges arising from aerial viewpoint geometry in aerial image-based adversarial robustness research. To the best of our knowledge, no existing review specifically examines how adversarial patch attacks affect UAV-based aerial object detection and challenges arising from aerial viewpoint geometry. The review by Chen and Chu [25] is the closest to ours. It examines the adversarial robustness of aerial detection, grouping digital and physical attacks by sensing modality, namely optical, infrared, and synthetic aperture radar imaging, and grouping defenses by the operational phase in which they are applied. Table 1 compares this review with existing surveys across four properties, where “viewpoint geometry” denotes an analysis of state-of-the-art studies based on how they integrate aerial viewpoint conditions, particularly altitude and look-down angle, rather than treating these factors solely as aspects of scale.

A central observation of this review is that aerial adversarial patch attack research has largely treated viewpoint variation as a scale variation problem. This assumption is reasonable for the near-nadir imagery that dominates existing benchmarks, but it becomes increasingly restrictive under oblique viewing conditions where visible surfaces, projection geometry, and physical degradation also change. This paper examines the extent to which this assumption influences current attack and defense methods. The main contributions of this paper are as follows:

- Analysis of the existing literature on adversarial patch attacks and defense methods for aerial object detectors, and release of the extracted per-paper corpus in a public repository.
- A unified taxonomy and comparative analysis of adversarial patch attacks, including their advanced forms, such as camouflage-based attacks, is presented, with a focus on the unique geometric challenges of aerial environments.
- A comprehensive analysis of existing defense mechanisms against adversarial patch and camouflage attacks in aerial object detection systems is presented.
- The widespread assumption that viewpoint variation can be represented through scale variation is critically analyzed, along with its limitations under oblique viewing conditions.
- Key challenges, research gaps, and future directions for aerial adversarial attack and defense research are discussed.

**Review Scope:** This review considers research on adversarial patch and camouflage attacks, together with corresponding defense mechanisms, for object detection in aerial, UAV, and remote-sensing imagery. The adversarial robustness of perception tasks such as segmentation using aerial imagery was excluded from this review, as the majority of the studies focused on object detection. The discussion focuses primarily on physically realizable attacks and the related unique challenges encountered when developing such attacks in the aerial imagery domain. Background information on adversarial examples, patch optimization, expectation over transformation (EoT), and object detection is also presented to provide knowledge of the initial concepts. Ground-level studies are also included in the review when aerial methods build on their techniques. Each study is categorized based on whether it was tested using aerial imagery, in simulation, or only at ground level. This makes it clear which findings are directly relevant to aerial applications. The resulting review is organized according to attack modality, target domain, and defense strategy, forming the basis of the taxonomy and analysis presented in the following sections.

The remainder of this paper is organized as follows. Section 2 outlines the criteria used to select the state-of-the-art studies included in the review. Section 3 then summarizes background information on object detection, adversarial patches, and their physical realization, providing the foundation for the subsequent sections. Next, Section 4 presents the proposed taxonomy of adversarial patch research on aerial object detection, while Section 5 compares existing methods and results. Section 6 then critically discusses the field, outlines the limitations and research gaps identified, and highlights the challenges that constrain further progress. Section 7 translates those gaps into future research directions, and finally Section 8 concludes the paper.

## 2. Literature Selection Criteria

To identify relevant articles for the review, Google Scholar was used as the primary search engine, while publications indexed in IEEE Xplore, the ACM Digital Library, ScienceDirect, Springer, Scopus, and Web of Science were considered for the period from January 2017 to 15 July 2026. The search combined four blocks of terms. The first block described the perturbation and used adversarial patch, adversarial camouflage, adversarial texture, and physical adversarial attack. The second described the task and used object detection, detector, and recognition. The third block described the domain and used aerial, UAV, drone, remote sensing, and satellite. The fourth described the countermeasure and used adversarial defense, patch defense, certified defense, and patch detection. Terms within each block were joined with OR, and the blocks were joined with AND. The terms in the domain block were omitted in some instances to retrieve ground-level work on which aerial methods are built. Furthermore, relevant articles were identified through forward and backward reference searching. Once relevant works were identified, their titles, abstracts, and conclusions were reviewed, and the relevant works were filtered based on the following criteria:

- Does it propose or evaluate physically realizable adversarial attacks, such as printed patches, 3D surface textures, or non-contact optical perturbations, or propose defenses against such attacks?
- Does the article evaluate such attacks or defense methods on aerial-image-based object detectors?

From this process, 107 papers were finalized for the review. For each retained paper, we recorded the thematic cluster, title, year, authors, venue, objective, method, datasets, detector, metrics, key result, limitations, relevance to viewpoint geometry, and source link. The complete corpus, together with these extracted fields, is published in a public repository listed in the Data Availability Statement.

Table 2 reports how the 107 retained papers are distributed across thematic clusters. The comparative tables in Section 4, Section 5 and Section 6 do not list all 107 papers. Those tables present representative methods, selected because this review analyses them in detail and because their reported conditions could be established from the source. The remaining papers support the review in two other ways. They are cited in the narrative sections and counted in corpus-level statements, such as the architecture distribution in Section 5.5 and the classification of limitations in Section 6.6. The repository holds the full record for every paper.

These retained studies are not all evaluated in the same way. Some are tested using real aerial images. Others are relevant to aerial imagery only because their attack mechanism could work in an aerial setting. To avoid treating these as equivalent, each work is classified using the following four labels, based on the strongest evidence reported:

- **R–Real aerial:** Tested using real aerial or remote-sensing images.
- **S–Simulated aerial/3D:** Tested only in a simulation or 3D environment, with no real aerial images used.
- **G–Ground level:** Tested using real ground-level images, with no aerial evaluation reported.
- **T–Transferable but untested:** Not tested in an aerial setting. Its relevance to UAVs is inferred from the attack mechanism.

These labels are used throughout the remainder of the article when analyzing the extracted literature. A claim about aerial behavior is supported only by studies labeled ‘R’ or ‘S’. Studies labeled ‘G’ or ‘T’ are included because aerial methods build on them, and they are identified as such wherever they are discussed.

## 3. Background

This section reviews adversarial attacks and defenses in UAV-based object detection, providing the basis for a subsequent geometric analysis of this trend.

### 3.1. Object Detection

Deep object detectors are commonly grouped into four families, and the differences between them matter for how a patch attack is formulated [26].

Two-stage anchor-based detectors, such as Faster R-CNN [27], first generate a set of region proposals and then classify each proposal. One-stage anchor-based detectors, such as SSD [28] and the YOLO releases up to YOLOv7 [29], remove the proposal stage. They predict bounding boxes directly against a dense set of predefined anchors. These YOLO releases add an explicit objectness score at each location and multiply it by the class score, whereas SSD reserves one class for the background instead of scoring objectness separately. Anchor-free detectors, such as FCOS [30] and CornerNet [31], also remove the anchor set. They predict object locations directly through centreness maps or keypoint heatmaps, and they are often preferred for the small targets that dominate aerial imagery. The YOLO family spans both groups. YOLOv8 and the later releases use an anchor-free head that predicts a class score and a box at every location of a feature grid, without a separate objectness term [32]. The family name therefore does not fix the head design, and the two must be distinguished when patch attacks are compared. A more recent group of end-to-end detectors treats detection as a set-prediction problem on a transformer backbone. These models remove the anchor set and the score-and-suppress stage together.

The reviewed literature does not cover these families evenly. YOLO variants [29] are the most frequent target by a wide margin, and the reported results cover both the anchor-based releases and the anchor-free ones. Faster R-CNN [27] and SSD [28] are the next most commonly targeted detectors. Transformer-based and set-prediction detectors appear in only a small number of studies, and no reviewed study reports results on FCOS or CornerNet. Section 5.5 gives the number of adversarial patch studies reported for each architecture and discusses the extent to which the reported evidence can be generalized.

### 3.2. Adversarial Examples and the Patch Threat Model

Deep neural networks can be induced to make errors through carefully constructed input perturbations [33,34]. Classic adversarial examples modify the entire image while constraining the perturbation under an $L_{p}$ norm, often using optimization methods such as projected gradient descent [35]. Although highly effective in digital settings and spread across the entire image, such perturbations are far less physically realizable because they are largely destroyed when the scene is printed, illuminated, and captured again by a camera.

Adversarial patches relax the norm constraint in exchange for spatial locality. Large perturbations are permitted within a small contiguous region, while the remainder of the image remains unchanged [2]. This trade-off enables physical realization because the perturbation can be printed and deployed in a scene, but it can be more perceptible in some cases, primarily due to unusual texture patterns. Patches are commonly categorized according to whether they are universal or image-specific, whether they suppress an existing detection or induce a false one, and whether they are generated under white-box or black-box transfer-based threat models [36].

More formally, let *f* denote the detector, *x* the input image, and δ the adversarial patch. A placement transform t∼T defines the location, scale, rotation, and photometric changes that the patch undergoes within the scene, together with the binary mask $M_{t}$ that marks the pixels it occupies. The attacked image is given by

(1) $$x_{\mathrm{adv}}=\left(1-M_{t}\right)⊙x+M_{t}⊙T_{t}\left(\delta \right),$$

where ⊙ is the element-wise product. The patch is then obtained by minimizing a detection loss over the image and transform distributions, regularized by a non-printability term and a total-variation term. This is the objective used by Thys et al. [3], with the non-printability score of Sharif et al. [37],

(2) $$\delta^{⋆}=\arg\min_{\delta }\;E_{x∼X,\;t∼T}\left[L_{\det}\left(f(x_{\mathrm{adv}})\right)\right]+\lambda_{\mathrm{nps}}L_{\mathrm{nps}}\left(\delta \right)+\lambda_{\mathrm{tv}}L_{\mathrm{tv}}\left(\delta \right),$$

where $L_{\det}$ is the detection loss the attacker suppresses, $L_{\mathrm{nps}}$ penalizes colors a printer cannot reproduce, $L_{\mathrm{tv}}$ penalizes abrupt changes between neighboring pixels, and $\lambda_{\mathrm{nps}}$ and $\lambda_{\mathrm{tv}}$ weight the two terms.

Adversarial camouflage is the same attack carried out over a surface rather than a plane, and is best read as an extension of patch attacks and Equation (2) rather than a separate threat model. The optimization variable becomes a texture τ defined over the surface of a three-dimensional model *S*. The step that places the patch into the image in Equation (1) is replaced by a differentiable renderer *R* that takes the viewpoint θ and the environment *e* as arguments,

(3) $$\tau^{⋆}=\arg\min_{\tau }\;E_{\theta ∼\Theta ,\;e∼E}\left[L_{\det}\left(f(R(S,\tau ,\theta ,e))\right)\right].$$

Equation (3) generalizes the optimization in Equation (2) by optimizing a surface texture over varying viewpoints and environmental conditions. Given this, a planar patch can be viewed as a special case of camouflage, where the attack is applied to one face of the object and changes in viewing geometry are represented by the transformation distribution T rather than explicitly rendered [7,8]. The practical consequence of these two approaches is a trade-off, where camouflage provides greater robustness across different viewpoints but requires a 3D model, a renderer, and reliance on simulation. This trade-off is discussed further in Section 4.3.

### 3.3. Physical Realizability

Deploying an adversarial patch in the physical world exposes it to conditions that are not encountered during digital optimization. These include different lighting conditions, variations in camera distance and viewing angle, motion blur, sensor noise, and color shifts introduced during printing. To improve the robustness of the adversarial patch, these conditions must therefore be anticipated during optimization. EoT addresses this challenge by optimizing the patch over a distribution of geometric and photometric transformations, including rotation, scale, and brightness variations [38]. In the notation of Equation (2), EoT is what the expectation over T stands for. Additional constraints are commonly incorporated to improve real-world applicability and robustness. A non-printability score penalizes colors that cannot be reliably reproduced by a printer [37], while a total variation penalty encourages smooth and physically realizable patterns. A typical physical attack objective therefore combines a detection-based loss with these two constraints, as given in Equation (2).

Early research involving road signs and object detectors established that carefully optimized physical patch attacks can remain effective after printing and image capture [39,40]. Most aerial adversarial methods inherit this formulation and primarily extend it to accommodate the large range of object scales encountered in UAV imagery. Figure 2 contrasts the digital and physical settings and illustrates the transformations that a printable patch must withstand, which are the transformations EoT is designed to cover.

### 3.4. The Aerial and UAV Setting

In computer vision research, the aerial setting introduces several unique challenges that are not present in the same way in ground-level imagery. UAVs can operate at different altitudes, causing substantial variation in object scale, with objects often occupying only a small portion of the image. The camera may also capture scenes from near-nadir to strongly oblique viewpoints, changing the visible surfaces and the degree of perspective distortion and foreshortening. Operational constraints add to these challenges. Detection frequently runs on embedded hardware with limited computational and energy resources, constraining both detector complexity and the feasibility of defense mechanisms. Aerial platforms also commonly employ thermal and multimodal sensors in addition to visible-spectrum cameras, resulting in a distinct body of research on adversarial attacks [10,12].

A recurring limitation within the literature concerns the availability of geometric information. Camera intrinsics, altitude, and pose are often readily available in simulation environments but are unavailable in most of the real-world aerial benchmarks [4,6,41]. Figure 3 illustrates this limitation. While real aerial imagery provides realistic visual content, commonly used benchmarks often lack the information needed to recover the viewing geometry. In contrast, simulators and calibrated multi-camera benchmarks such as Wildtrack [42] provide exact geometric information but cannot fully establish real-world validity. Camouflage attacks illustrate this limitation. FCA [7], a camouflage attack on vehicle surfaces, varies camera elevation, azimuth, and distance in CARLA [43], while DE-DAC [44], another camouflage-based method, controls camera rotation and location through its neural renderer.

Table 3 summarizes existing datasets and simulations used in aerial-imagery research and highlights the limitation discussed above. Specifically, most real aerial datasets, including VisDrone [41], DOTA [45], DroneVehicle [46], and CARPK [47], lack camera intrinsics, altitude, and pose. Such geometric information is available mainly in calibrated or simulated environments. For example, Wildtrack [42] provides calibrated extrinsics, while simulators such as CARLA [43], Unreal Engine [48], and AirSim [49] provide parameters such as camera intrinsics, altitude, and pose through their programming interfaces.

A limited number of real aerial imagery datasets provide flight metadata. SeaDronesSee [50] records per-frame altitude and gimbal pitch, while UAVDT [51] and HIT–UAV [52] provide coarse altitude and camera angle information. Confirming the challenges that this review focuses on, HIT–UAV [52] reports that detection precision initially increases and then decreases with increasing camera angle, attributing this trend to the shrinking visible surface of the target rather than to scale. Furthermore, EVD4UAV [53], a real-world dataset specifically designed for adversarial patch attacks against vehicle detection, also provides flight-altitude information, although its camera remains near-vertical throughout.

More broadly, altitude and other camera-extrinsic and intrinsic information can enter a study in three main ways: (i) they can be used to group results by altitude or imaging conditions; (ii) they can be used to scale the perturbation according to flight height; or (iii) they can be incorporated into a geometric model that reasons about projection, visible surfaces, and perturbation placement. Existing studies, such as EVD4UAV [53], primarily use altitude for (i) and (ii). In contrast, this review did not identify a study in the corpus that incorporates altitude or other aerial-imaging camera parameters into an explicit geometric model for adversarial patch research, which is unsurprising because such information is not available in most of the aforementioned real-world datasets.

**Table 3:** Evaluation datasets and simulators used in the reviewed literature. Geometry column: *A* altitude, *P* camera pose, *K* camera intrinsics. A superscript ∗ marks a parameter that is set inside a simulator or renderer rather than measured, and a superscript † marks partial information, such as binned altitude or gimbal pitch alone. 🞬 marks a dataset that supplies none of the three. Not identified in use by any attack in the corpus is represented by —.

Dataset	Domain/Modality	Viewpoint	Geometry	Used By
VisDrone [41]	UAV RGB, 10 classes	Oblique + nadir	🞬	URAdv [6], CDUPatch [12], Cui et al. [54].
DOTA [45]	Aerial RGB, 15 classes	Overhead	🞬	AP-PA [4], Lu et al. [55].
RSOD [56]	Aerial RGB, 4 classes	Overhead	🞬	AP-PA [4], Lu et al. [55].
NWPU VHR-10 [57]	Aerial RGB, 10 classes	Overhead	🞬	Lu et al. [55].
DroneVehicle [46]	UAV RGB + infrared	Oblique	🞬	CDUPatch [12], EMA [58].
CARPK [47]	Drone RGB, cars (1 class)	Near-nadir	🞬	EMA [58].
MSDrone [12]	UAV RGB + IR, multi-scale	Varied	🞬	CDUPatch [12].
COWC [59]	Aerial RGB, 1 class	Overhead	🞬	Digital-to-Physical [60].
LLVIP [61]	Low-light surveillance for pedestrian detection (RGB + thermal IR)	Ground-level oblique	🞬	CDUPatch [12].
SeaDronesSee [50]	UAV RGB, maritime search and rescue	5–260 m, 0–90 degrees	*A*, *P*^†^	—
HIT–UAV [52]	UAV thermal IR, persons and vehicles	60–130 m, 30–90 degrees	*A*^†^, *P*^†^	—
EVD4UAV [53]	UAV RGB, vehicles; built for patch attacks	Near-nadir, 50/70/90 m	*A* ^†^	Sun et al. [53] (baseline patch attacks).
UAVDT [51]	UAV RGB, vehicles	Front/side/bird	*A*^†^, *P*^†^	—
Wildtrack [42]	Multi-camera pedestrian detection	Multi-view ground	*P*, *K*	Fool the Hydra [62].
CARLA [43]	Driving simulator, RGB	Controllable	*A*^∗^, *P*^∗^, *K*^∗^	FCA [7], MOA [63].
Unreal Engine 4 scenes [48]	Rendering engine; persons and vehicles in three outdoor scenes	Controllable	*A*^∗^, *P*^∗^, *K*^∗^	DE-DAC [44].
AirSim [49]	UAV and vehicle simulator built on Unreal Engine; RGB, depth, and segmentation	Controllable, full flight envelope	*A*^∗^, *P*^∗^, *K*^∗^	—

### 3.5. Evaluation Metrics

Attack and defense performance on aerial object detection networks are typically evaluated using a set of metrics, as summarized in Table 4. These metrics cover bounding-box detection only, and mask average precision (AP) and other segmentation measures are outside the scope stated in Section 1. Detection performance is most commonly reported using AP or mean average precision (mAP) at one or more intersection-over-union (IoU) thresholds. Attack effectiveness is measured either through the corresponding reduction in these metrics or directly through attack success rate (ASR), defined as the proportion of targets successfully suppressed or misclassified. Precision and recall are frequently reported to provide additional insight into detector behavior, while multi-view detection literature often employs multi-object detection accuracy (MODA).

Defenses are evaluated with the same detection metrics rather than with a separate family of measures. The usual protocol reports the metric twice, once on clean inputs and once on attacked inputs, so that a defense is judged both on the detection performance it restores under attack and on the performance it costs when no attack is present. Certified defenses report an additional quantity, namely the proportion of inputs for which robustness is guaranteed against any perturbation within a stated threat model [16,64,65,66]. The last two rows of Table 4 record these two cases.

## 4. Classification of Existing Research

### 4.1. A Taxonomy of the Field

The literature on adversarial patch research can be organized along two axes: (1) the medium in which the adversarial perturbation is applied and (2) the application domain it targets. Five notable techniques appear across the reviewed studies, namely planar printed patches, textures wrapped over three-dimensional surfaces, deformable fabrics worn on the body, thermal and dual-modal perturbations, and non-contact optical perturbations projected into the scene. The domains include generic detection benchmarks, ground-level pedestrian and vehicle scenes, aerial and remote-sensing imagery, and multi-camera systems. Table 5 summarizes the representative studies based on these two axes and records the strongest evaluation setting reported for each work using the aerial relevance classification presented in Section 2. We list ground-level work alongside aerial work because aerial methods use their losses, EoT metrics, and printability constraints originally developed for ground-level work.

Defenses are classified differently because they do not have their own perturbation medium. Instead, they are classified by the medium they are designed to resist and the domain in which they were evaluated, and they are indexed on those two properties in the final block of Table 5. This distinction is important because, unlike the attack literature, most defense methods in the corpus assume a planar patch viewed in a ground-level image. This limitation is revisited in Section 4.6 and identified as an existing research gap in Section 6.5.

Figure 4 narrows the same organization to the UAV setting and separates the dimensions. Level 1 represents the deployment mechanism, distinguishing perturbations that are physically attached to the target from those delivered remotely. Level 2 represents the physical medium and follows the five media defined in Table 5, in the same order. Level 3 characterizes the perturbation according to its spatial support, sensing modality, and the evidence supporting its relevance to the aerial imagery domain, as defined in Section 2. Distinguishing deployment mechanism from sensing modality is particularly important for thermal and dual-modal carriers. A dual-modal patch is physically printed and attached to the target, making it a contact attack, even though its effect is directed at the infrared channel. Classifying such attacks solely by sensing modality would group them with lasers and projected light, even though the latter reach the target through non-contact mechanisms. Accordingly, CDUPatch [12] is classified under the thermal and dual-modal categories on the contact side of the taxonomy, whereas the non-contact category contains optical projection attacks.

The evidence supporting the relevance of each attack to aerial imagery research varies substantially across perturbation media. The planar branch has been evaluated using real UAV and remote-sensing imagery, whereas the 3D surface branch has been evaluated predominantly in rendered environments. Deformable fabric attacks have been demonstrated only from ground-level viewpoints, while optical projection currently lacks evidence from aerial platforms. Similarly, laser, projected-light, shadow, and raindrop attacks have been evaluated using cameras observing targets from short-range, ground-level viewpoints. These attacks are included in the taxonomy because UAV-mounted cameras are, in principle, susceptible to the same perturbations, rather than because they have been experimentally demonstrated in aerial settings. Section 4.4 discusses the factors that currently limit such aerial validation. The thermal and dual-modal branch represents the only mixed-evidence category, combining CDUPatch, which has been evaluated using real aerial imagery, with other infrared-based approaches demonstrated exclusively at ground level.

Based on this, it is evident that two of the five media receive the most attention. The first is planar printed patches applied to aerial and remote-sensing imagery, where viewpoint variation is handled through scale adaptation. The second is three-dimensional camouflage, where viewpoint variation is addressed through geometry and rendering. Section 4.2 and Section 4.3 therefore treat these two media in detail. The remaining three media, namely deformable fabric, thermal and dual-modal perturbations, and non-contact optical perturbations, are treated more briefly in Section 4.4, because most of the work using them is evaluated at ground level and carries less direct evidence for the aerial case.

**Table 5:** A two-axis taxonomy of adversarial patch and camouflage research: perturbation medium against application domain. The evidence column follows Section 2, and only ‘R’ and ‘S’ rows support claims about aerial behavior.

Medium	Application Domain	Defining Trait	Evidence	Representative Works
Planar printed patch	Generic detection and classification	The original patch formulations: a printed pattern optimized against a classifier or a detector without a specific scene in mind.	G	Brown et al. [2]; DPatch [36].
Planar printed patch	Road scenes and vehicles, ground level	Patches printed for signs or vehicle bodies and evaluated from a driver’s-eye viewpoint.	G	RP2 [39]; Eykholt et al. [40]; Zhu et al. [82].
Planar printed patch	Pedestrians, ground level	Person-detector suppression, including the naturalistic line that constrains how the pattern looks.	G	Thys et al. [3]; Hu et al. [83]; Lapid et al. [84]; AdvART [85]; DAP [86].
Planar printed patch	Aerial, UAV, and remote sensing	Small, densely packed targets seen from above; scale adaptation is the main addition to the ground-level recipe.	R	AP-PA [4]; RSI MultiScale [5]; URAdv [6]; Lu et al. [55]; Tang et al. [87]; den Hollander et al. [88]; Du et al. [89]; Cui et al. [54]; PatchGen [67]; Digital-to-Physical [60]; EMA [58]; Rust-Style Patch [68].
Planar printed patch	Multi-camera and multi-UAV systems	A patch that must survive fusion across several calibrated views rather than defeating one camera.	G	Fool the Hydra [62]; UCDNet [90].
3D surface texture (camouflage)	Vehicles in driving simulators	A texture optimized over the whole vehicle surface through a differentiable or surrogate renderer.	S	FCA [7]; DAS [8]; DTA [9]; CAMOU [71]; UPC [72]; CNCA [91]; SC-PCA [92].
3D surface texture (camouflage)	Human and vehicle models in rendered scenes	Full-surface texture on a 3D human or vehicle mesh, optimized against a rendered background.	S	MOA [63]; DE-DAC [44]; MeshAdv [93].
Deformable fabric (wearable)	Pedestrians, ground level	Garments carrying an adversarial pattern, with cloth deformation modelled during optimization.	G	Adversarial T-shirt [73]; AdvTexture [74]; AdvCaT [75].
Thermal and dual-modal	Pedestrians and vehicles, ground level	Perturbation of the heat signature rather than the visible appearance.	G	Wei et al. [10]; Zhu et al. [11]; HotCold Block [76]; Hu et al. [94]; Dong et al. [95]; Li & Hao [96].
Thermal and dual-modal	Aerial and UAV	One pattern that has to work in the visible and infrared channels of a dual-modal UAV detector at once.	R	CDUPatch [12].
Non-contact optical	Classification and road scenes, ground level	Light, shadow, reflection, or weather projected into the scene, with nothing attached to the target. No aerial demonstration was identified. Section 4.4 discusses why flight makes one difficult.	T	AdvLB [77]; RFLA [78]; AdvRain [79]; Zhong et al. [80]; Gnanasambandam et al. [81].
Defense (built against planar patches)	Generic and ground-level detection	Localization and restoration, certified masking, and input pre-processing, all assuming a compact printed region and a fixed viewpoint.	G	PatchGuard [16]; ObjectSeeker [65]; PatchCleanser [66]; SAC [15]; Jedi [97]; PAD [98].
Defense (built against planar patches)	Aerial and UAV; embedded platforms	The small body of work that targets the UAV case or the hardware it runs on.	R for [14]; G otherwise	Pathak et al. [14]; PatchBlock [99]; quantization robustness [100].

### 4.2. Patch Attacks on Aerial and Remote-Sensing Detection

Adversarial patches developed for aerial and remote-sensing imagery constitute the central focus of this review. Early studies demonstrated the feasibility of physically realizable attacks against aerial object detectors and established many of the design principles adopted by subsequent work [88,89]. Since then, most methods have converged on a common optimization framework consisting of detector-confidence suppression, EoT, and printability constraints [4,5,6].

Figure 5 illustrates this shared pipeline. Specifically, it demonstrates a white-box attack pipeline because the gradients used to update the patch pass through the detector, meaning that the attacker is assumed to have access to the model’s weights. Black-box variants replace these gradients in one of two ways. The first is query-based search over the patch pixels, for example through differential evolution against aerial detectors [101] or through query-budgeted adaptive search [102]. The second is the use of one or more surrogate detectors, where gradients are taken through a model the attacker holds, and the resulting patch is expected to transfer to the deployed model [103,104]. These settings are discussed further in Section 4.5. During optimization, the patch is transformed, composited into aerial imagery, evaluated with a detector, and updated using gradients derived from objectness or confidence-suppression losses. The principal adaptation for aerial imagery is maintaining effectiveness across varying target scales, which the pipeline addresses through a scale-adaptation branch. The pipeline does not decide where on the object the patch is placed, and it samples physical degradations without reference to the viewing angle. Section 5.3 and Section 6.4 address both points.

Scale adaptation is the mechanism on which nearly all of this work relies, including area-ratio scaling [4], altitude-dependent scaling [5], nested multi-scale structures [6], and object-specific dimension adjustment [55]. Related remote-sensing studies extend similar ideas through universal perturbations, context-oriented attacks, visual-consistency optimization, and feature-aware formulations [69,70,105,106,107,108,109,110]. The implications of this choice are examined in Section 5.3 and Section 6.2.

### 4.3. Three-Dimensional and Multi-View Camouflage

Three-dimensional camouflage approaches handle viewpoint variation differently. Rather than optimizing a localized planar patch, these methods optimize textures that cover substantial portions of an object’s surface and explicitly model viewpoint changes via rendering and geometric transformations [7,8]. Equation (3) formalizes this approach.

Representative studies apply full-surface adversarial camouflage textures to vehicles, pedestrians, and other objects using differentiable rendering pipelines [7,8,9,44,63]. Earlier work established several key components. These include perturbation of the mesh geometry, meaning modification of the shape of the three-dimensional model rather than only the texture painted on it [93], together with surrogate rendering models and transformation-aware optimization [71,72]. More recent studies have placed greater emphasis on realism, environmental consistency, and perceptual plausibility [91,92,111].

Figure 6 illustrates the distinction between planar patch and adversarial camouflage attacks. As an object rotates, a localized patch may become increasingly distorted or move outside the visible region, whereas a surface texture can maintain adversarial coverage across multiple viewpoints. These methods can improve robustness to viewpoint variation, but often require an accurate 3D model, a differentiable rendering pipeline, and extensive simulation-based optimization. Evaluation is conducted primarily in rendered environments such as CARLA and Unreal Engine, where viewpoint geometry is precisely controlled. Consequently, a key challenge is reducing reliance on simulation while maintaining robustness to viewpoint variation.

The viewpoint coverage reported in this line of work is also narrower than the term “multi-view” may suggest. FCA varies camera elevations from 0 to 50 degrees in CARLA [7], so its multi-view evaluation covers oblique viewpoints and does not extend to near-nadir viewpoints. To the best of our knowledge, none of the camouflage studies reviewed here reports results at the near-vertical viewing angles relevant to UAV operation. This observation is limited to the studies retrieved and reviewed in this work and is not intended to generalize to the broader camouflage literature. Whether textures optimized and evaluated over this elevation range remain effective when the camera approaches a near-nadir viewpoint therefore remains an open question. This limitation provides one basis for distinguishing the existing camouflage literature from the aerial patch literature.

### 4.4. Naturalistic, Infrared, and Optical Stealth Attacks

A parallel line of research prioritizes attack stealthiness over maximum effectiveness, aiming to reduce visual or physical conspicuity while maintaining adversarial performance. Naturalistic attacks constrain the perturbation to resemble realistic textures or scene content. Three mechanisms are used. The patch may be restricted to the output range of a pretrained generative model, so that every candidate pattern is drawn from a distribution of natural images [83,84]. A similarity term may be added to the objective, penalizing the distance between the optimized patch and a chosen reference image, measured either in pixel space or in the feature space of a perceptual network [85]. A style-transfer objective may be used to impose the appearance of a reference texture on the patch [68]. Related aerial work investigates camouflage patterns that blend into overhead imagery through texture design and environmental adaptation [68,112,113].

Infrared patch attacks target thermal sensing systems by manipulating heat signatures rather than visible appearance [10,11]. Subsequent work has explored learnable thermal structures, wearable thermal perturbations, multi-view infrared attacks, and cross-modal transfer between visible and thermal domains [12,76,94,95]. Li and Hao [96] take this line furthest towards a deployable system. They optimize an optical patch against YOLOv5 and an infrared patch by differential evolution over its shape, then combine the two on an indium tin oxide film that is transparent in the visible band and reflective in the infrared, so that a single physical article attacks both channels at once. The attack is physically realized and reduces detector mAP by more than 75%, but neither patch is conditioned on the viewing geometry, and the evaluation is conducted at ground level against static backgrounds.

Optical attacks eliminate the need for physical surface modification by projecting perturbations into the scene using lasers, projected light patterns, shadows, reflections, atmospheric effects, or infrared sources [77,78,79,80,81,114,115,116].

These attacks are carried out at the evidence level ‘T’. To the best of our knowledge, no study in the corpus demonstrates a laser, projected-light, shadow, or raindrop attack against a camera in an aerial configuration. Three features of flight work against a direct port from ground-level demonstrations. The first is standoff distance. The reported laser and projected-light demonstrations operate over meters, whereas a UAV observing a ground scene typically sits tens to hundreds of meters away. Irradiance from a divergent source falls with the square of that distance. Ambient illumination compounds the problem because a projected pattern has to compete with daylight on the target surface. The second is registration. A projected perturbation has to land on a particular part of the image, and the relative pose of the aircraft, emitter, and target changes continuously under airframe vibration, gimbal motion, and target movement. The pointing tolerance is therefore far tighter than for a static ground rig. The third applies to attacks that act on the optical path rather than on the scene, such as adhesive raindrop films [79]. The lens sits inside a gimbal housing on the aircraft, which an attacker on the ground cannot reach.

None of this rules out the medium. An emitter mounted on the target rather than remote from it would face milder versions of these constraints, as would an attack aimed at a low-altitude or dawn-and-dusk operating envelope. The point is that their aerial feasibility is untested. They are listed in the taxonomy for completeness of the threat picture, labeled ‘T’ throughout, and none of the conclusions this review draws about aerial attack behavior rests on them.

Although these approaches broaden the range of attack modalities, most are developed and evaluated in ground-level settings. Consequently, their assumptions about concealment, naturalness, and visibility may not translate directly to UAV imagery.

### 4.5. Existing Research Directions in Attack Design

Two themes are prominent across perturbation media and application domains, namely loss-function design and transferability. Most detector-oriented attacks optimize for objectness or detection confidence rather than class probabilities. This strategy reflects the structure of modern detectors, in which preventing a detector from generating a bounding box is often more effective than manipulating its final classification decision. Most aerial patch methods adopt this formulation directly [4,5,6].

Recent studies have revisited the optimization process itself along several lines. These include patches that adapt during the attack rather than being fixed after training [86], spectral and semantic regularization applied to intermediate features to improve transfer from vision transformers [117], diffusion priors used to generate the patch pattern [118], ensemble objectives combined with a lightweight visual-similarity term that improves transferability and reduces conspicuity without an additional generative model [119], prompt-guided objectives that keep the pattern consistent with the scene it is placed in [120], and objectives designed for perception tasks other than detection, such as monocular depth estimation [121].

Transferability is the second theme. Because attackers rarely have access to the weights of a deployed detector, considerable effort has focused on developing attacks that generalize across architectures. In a black-box setting, the attacker cannot access the deployed model’s weights or backpropagate through it. Instead, the attacker must rely either on the model’s outputs for a set of probe inputs or on a separately trained surrogate model, with the expectation that the resulting perturbation will transfer to the target model. This represents a realistic threat model for a deployed UAV detector and is also the more challenging setting, which may explain why many reported ASRs are still measured under white-box conditions [82,101,102,103,104,122,123,124].

Wearable attacks constitute a specialized application of these principles, addressing the additional challenge of deformable surfaces through fabric deformation models, tiled textures, and body-aware optimization frameworks [73,74,75]. Although the implementation differs, the underlying goals of robustness and transferability remain the same.

### 4.6. Defense Methods

Defenses against adversarial patches fall into four families. The first and most extensively studied family comprises patch detection and restoration, which localizes the perturbed region and then restore the affected area. The adversarial patch is first localized using gradient, entropy, or saliency cues and then masked or zeroed out. The missing content is subsequently reconstructed using inpainting or other generative networks [15,97,98,99,125,126,127,128,129]. These operations are best understood as components of a single pipeline rather than as competing methods, since localization alone does not recover the affected visual content. The second family comprises model-level robustness methods, which modify the detector or its training procedure rather than the input presented at inference time. Adversarial training belongs here, because it changes the training data and the resulting weights while leaving the deployed inference pipeline unchanged. Other examples include training the model to identify the patch as a class of its own [130] and studying how robustness is affected by quantization [100]. The third family, certified robustness, provides formal guarantees by constraining the model’s receptive fields, systematically masking possible perturbation regions, or otherwise bounding the influence of a localized perturbation [16,64,65,66,131]. The fourth family includes input pre-processing methods, which transform the image before it reaches the detector and do not attempt to reconstruct the affected content. Examples include local gradient smoothing, which suppresses high-frequency regions where a patch produces strong image gradients [132], outlier rejection and dimensionality reduction [133,134], and randomized or filtered input transformations [135]. These four families are consistent with the three-part scheme used in the broader adversarial-robustness literature, which classifies defenses into three categories: modifying the data, modifying the model, and using auxiliary tools [136]. Furthermore, a separate line of research examines robustness under deployment constraints, including large-scale benchmark evaluations and real-time protection strategies [137,138,139].

Table 6 outlines existing defense methods based on criteria relevant to their potential deployment on UAV platforms. These criteria include the target task, whether robustness is supported by formal certification or empirical evaluation, the perturbation medium against which the defense was evaluated, and whether computational cost has been reported for hardware representative of UAV platforms. Two of these criteria remain largely unreported. Every reviewed defense is evaluated against planar patches alone, and only three studies report computational cost. DetectorGuard [64] reports a per-module runtime breakdown and notes that a single-GPU deployment roughly doubles inference time. NutNet [139] reports an 8% increase in inference time. PatchBlock [99] was designed for embedded hardware. The remaining studies report neither throughput nor memory. None report how the defense behaves after quantization, which is the reduction of the weights and activations of a model to a lower numerical precision so that it fits the memory and power budget of an embedded board. Quantization is applied to the detector before deployment, so a defense that is robust at full precision may not stay robust on the board that actually flies. Only one reviewed defense, that of Pathak et al. [14], is evaluated on aerial imagery. The implications of these limitations are discussed in Section 6.5.

Figure 7 depicts a summary of these defense techniques. Despite the methodological diversity of the reviewed approaches, most defense methods are evaluated using ground-level imagery, static viewpoints, and computational resources that may not reflect the constraints of UAV deployment. Consequently, the interaction between viewpoint geometry, physical adversarial perturbations, and the computational constraints of UAV platforms remains underexamined.

**Table 6:** Structured comparison of the defenses in the corpus. “Not reported” means the source does not state the quantity, and a dash means the field could not be established from the record. In the last column, 🞬 marks neither setting, 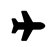 marks evaluation on aerial imagery, and 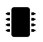 marks evaluation on embedded hardware.

Defense	Family	Task	Guarantee	Attack Medium Evaluated	On-Board Cost Reported	Aerial or Embedded Evaluation
PatchGuard [16]	Certified robustness	Classification	Certified	Planar patch	Not reported	🞬
PatchCleanser [66]	Certified robustness	Classification	Certified	Planar patch	Not reported	🞬
DetectorGuard [64]	Certified robustness	Detection	Certified	Planar patch	Yes. Per-module runtime breakdown; roughly 2x slower on a single GPU	🞬
ObjectSeeker [65]	Certified robustness	Detection	Certified	Planar patch	Not reported	🞬
SAC [15]	Detection and restoration	Detection	Empirical	Planar patch	Not reported	🞬
Jedi [97]	Detection and restoration	Detection	Empirical	Planar patch	Not reported	🞬
PAD [98]	Detection and restoration	Detection	Empirical	Planar patch	Not reported	🞬
DIFFender [128]	Detection and restoration, diffusion prior	Detection	Empirical	Planar patch	Not reported	🞬
Ad-YOLO [130]	Model-level robustness	Detection	Empirical	Planar patch	Not reported	🞬
Guesmi et al. [100]	Model-level robustness, quantization study	Detection	Empirical	Planar patch	Quantized models studied. Latency and memory not reported	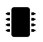
PatchBlock [99]	Detection and restoration, lightweight	Detection	Empirical	Planar patch	Yes. Developed for embedded EdgeAI hardware	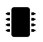
Pathak et al. [14]	Model-agnostic input defense	Detection	Empirical	Planar patch	—	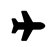
NutNet [139]	Detection and restoration, reconstruction prior	Detection	Empirical	Planar patch, hiding and appearing	Yes. An 8% increase in inference time	🞬
Wu et al. [137]	Unified re-evaluation of the above families	Detection	Empirical, comparative	Planar patch	—	🞬

## 5. Comparative Analysis

### 5.1. Basis for Comparison

This section compares the reviewed attacks in two stages. The methods are compared first by what each one models, including the loss it optimizes, how it treats altitude and viewing angle, where it places the perturbation, and what access to the detector it assumes.

The reported results are compared with existing methods for reference, but not ranked against them because they were evaluated under different settings. Specifically, studies report different metrics, including ASR, AP reduction, mAP reduction, and precision at fixed IoU thresholds. Furthermore, these metrics are computed on different datasets, detector architectures, perturbation sizes, and physical protocols. Section 3.4 outlines how the aerial benchmarks differ in what they record. The conclusions of this review are based on differences in attack formulation, viewpoint modeling, and evaluation design. Table 7 records what each study reports rather than providing a direct ranking of the methods. The performance measures reported by each method, whether ASR or a comparable metric, are listed in the reported performance column of that table. These measures are not standardized. Where ASR is reported, the definition of a successful attack differs between sources, so each value describes one study on its own terms rather than on a shared scale.

### 5.2. Methods and Mechanisms

Table 7 summarizes the attack methods most relevant to this review. Despite implementation differences, the aerial patch literature exhibits a remarkably consistent methodological approach. First, most approaches optimize variations of the same objective. AP-PA [4] suppresses detector objectness after non-maximum suppression, RSI MultiScale [5] aggregates confidence scores across detections, and URAdv [6] combines confidence and objectness terms within a unified loss. Subsequent methods largely refine this formulation rather than replacing it. Second, viewpoint variation is typically represented through scale adaptation. Existing approaches accommodate altitude changes using area-ratio scaling [4], height-dependent scaling [5], nested multi-scale patches [6], multi-scale clipping [12], or object-specific resizing strategies [55]. While these mechanisms differ operationally, they all assume that the principal consequence of altitude variation is a change in apparent object size. These mechanisms do not fully remove the effect of height. RSI MultiScale [5] keys its patch scale to the recorded capture height and still reports an attack success rate that falls from 96% at 25 to 40 m to 87.26% at 105 to 120 m on YOLOv3. Correct resizing therefore reduces the effect of altitude without eliminating it.

The notable exception is Cui et al.’s [54] projection-aware attack, which explicitly incorporates image projection into patch deformation. However, even this method adapts the shape of the patch rather than determining which face of the target it should occupy. The distinction matters because a vehicle viewed from a UAV cannot be represented as a single plane. It comprises several surfaces, including the roof, bonnet, windscreen, and side panels, whose visibility and apparent size vary with the look-down angle. At near-nadir angles, the roof dominates the visible area and is the natural carrier for a patch. As the camera moves towards the horizon, the roof occupies fewer pixels, while the side and rear panels become more visible. Cui et al. [54] show where the projection-aware approach stops. Four corner points of the vehicle roof are annotated by hand, and the patch is warped onto that roof quad using a homography recomputed for each viewing angle and distance. The patch therefore moves and deforms with the viewpoint, but it stays on the roof, because the annotation fixes the roof as the carrier before optimization begins. Choosing the surface as a function of the look-down angle is a step that was not identified in any of the reviewed methods.

**Table 7:** Attack methods analyzed in this review. WB and BB denote white box and black box. A downward arrow marks the reduction in a detector metric caused by the attack, so a larger value indicates a stronger attack. Reported performance is not comparable across rows, and a dash marks a field that could not be established from the record.

Method	Evidence	Threat	Objective	Loss Terms	Evaluation	Support and Scaling	Datasets	Detectors	Reported Performance	Transfer
AP-PA [4], 2022	R	WB, BB	Untargeted hiding	Objectness after NMS, TV, NPS	Digital, physical	Planar, on or outside target, fixed area ratio	RSOD, DOTA	YOLOv2/v3/ v5, SSD, Faster R-CNN, Swin	AP ↓ 87.86% WB, ↓ 85.48% BB	Yes, all listed detectors
RSI MultiScale [5], 2022	R	WB	Untargeted hiding	Mean object confidence, box, class, NPS	Digital, physical	Planar, on the car, scaled by capture height	Custom aerial, 25–120 m	YOLOv3, YOLOv5	ASR 92.65% (YOLOv3) and 60.16% (YOLOv5) at confidence 0.5	YOLOv3 to YOLOv5
URAdv [6], 2025	R	WB	Untargeted hiding	Confidence and objectness, TV, NPS	Digital	Planar, nested multi-scale, keyed to flight height	VisDrone-13	YOLOv5-S, YOLOv5-L	ASR 82.0% WB, 78.5% under transfer	YOLOv5-S to YOLOv5-L
CDUPatch [12], 2025	R	WB	Untargeted hiding	color-driven loss, visible and infrared	Digital	Planar, on target, multi-scale clipping	DroneVehicle, VisDrone, LLVIP, MSDrone	YOLOv3/v5/ v8, Faster R-CNN, dual-modal	ASR 57.5–70.4% on DroneVehicle, and 66.7–73.1% for YOLOv5 across four sets	Yes, black box on DroneVehicle
EMA [58], 2024	R	WB	Untargeted hiding, color-constrained	Confidence, objectness, TV, color prior	Digital, physical	Planar, 30% of vehicle area, ±20° rotation	CARPK, DroneVehicle	YOLOv5 l, x, and two UAV-trained	mAP within 2 points of the baseline. Physical mAP 27.4 against 30.8	Cross-dataset, four YOLOv5 victims
Digital-to-Physical [60], 2026	R	WB	Untargeted hiding	Maximum objectness, NPS, TV	Digital, physical	Planar. On vehicle, off in context, off to the side	COWC; Side Street, Car Park	YOLOv3	AORR 85.51% (off) against 67.43% (off-side). OSR 0.197–0.343 (on)	—
Cui et al. [54], 2024	R	WB	Untargeted and targeted	Max box confidence, adversarial distance, TV, NPS	Digital, physical	Planar, warped onto a hand-annotated roof quad	Custom UAV 10–30 m, VisDrone	YOLOv3, Faster R-CNN	ASR 38.50% and 20.83% on YOLOv3. Printed model 47.02%	Poor to Faster R-CNN, good to YOLOv5–v9
Lu et al./ PatchNoobj [55], 2021	R	WB	Untargeted hiding	Object-confidence suppression	Digital	Planar, universal, sized to the aircraft	DOTA, NWPU VHR-10, RSOD	YOLOv3, plus two under transfer	AP 93.1 → 44.9, 88.1 → 64.2, 92.0 → 71.8%	Across datasets, and to YOLOv5 and Faster R-CNN
FCA [7], 2022	S	WB, transfer	Untargeted hiding	Detection loss, smoothness, NPS	Simulation	Full vehicle surface, rendered, elevation 0–50°	CARLA	YOLOv3, and four more under transfer	P@0.5 ↓ 60% on YOLOv5, ↓ 52.04% on Faster R-CNN	Yes, four detectors
DE-DAC [44], 2023	S	WB, transfer	Untargeted hiding with stealth	Scene match, local texture, DE search	Simulation	Full surface, neural renderer	Unreal Engine 4, person, cars, trucks	YOLOv3, and four more under transfer	P@0.5 100 → 10.0–25.4%. ASR 74.6–90.0% over three scenes	Yes, four detectors
MOA [63], 2023	S	WB	Untargeted hiding, mimetic style	Mimetic style, adversarial, human-eye	Simulation	Full surface on a 3D human, background-conditioned	CARLA	YOLOv5	mAP@0.5 ↓ 67.62%, average ASR 85.28%	Yes, YOLOv7, SSD, Faster R-CNN
Fool the Hydra [62], 2023	G	WB	Untargeted hiding	Focal loss, cross-view gradients, attention term	Digital, multi-camera	Planar, optimized across calibrated views	Wildtrack	MVDet, MVDeTr	MODA ↓ 73.77% (MVDet) and ↓ 62.57% (MVDeTr), from two patches	MVDet patch fails on MVDeTr

### 5.3. Viewpoint and Geometry Handling

Table 8 compares attack methods across eight viewpoint-related dimensions. These include scale adaptation, projection modeling, whether camera pose is treated as an explicit quantity rather than absorbed into augmentation, surface selection, altitude metadata, viewing-angle metadata, viewpoint-stratified degradation, and physical validation. A clear pattern emerges. Scale adaptation is common and is the dominant mechanism by which aerial attacks address viewpoint variation. In contrast, explicit projection modeling appears only in Cui et al.’s [54] projection-transformation attack. Even there, geometry is used to deform the patch rather than to reason about target visibility.

Likewise, surface selection remains largely unexplored. Most methods assume a fixed placement region, typically on the roof or another manually selected surface. The recent digital-to-physical study [60] compares a patch attached to the vehicle body with a patch placed in the surrounding context and reports that the contextual patch is stronger in the digital domain, whereas the on-object patch is more effective after physical deployment. This comparison considers two fixed placement configurations, neither of which is selected as a function of the viewing geometry.

The altitude-metadata columns should be read alongside Section 3.4. EVD4UAV [53] and RSI MultiScale [5] both use a recorded height, but in both cases, the height determines a scale factor rather than which surface the patch occupies. The angle column is empty for all rows except the two camouflage methods, where the angle is a renderer setting rather than a measurement. The most striking observation concerns physical degradation modeling. Existing methods rely almost exclusively on generic transformation distributions, such as those used in EoT frameworks. We did not identify any study in the reviewed literature that conditions its degradation model on viewing angle or look-down geometry.

Figure 8 illustrates the difference between fixed placement against geometry-dependent placement. Placement conditioned on viewpoint geometry is underexplored, and two practical constraints account for part of the reason. The attacker has to recover the viewing geometry of a UAV it does not control, and a printed patch cannot be moved once it is fixed to the target. Section 6.6 sets out both constraints, and Section 7 treats them as a research direction.

Taken as a whole, the literature falls into three broad groups. Aerial patch methods model viewpoint primarily through scale adaptation while keeping placement fixed. Three-dimensional camouflage methods achieve viewpoint robustness through full-surface coverage and rendering-based optimization, but rely heavily on simulation. Projection-aware methods incorporate geometric transformations but do not explicitly account for viewpoint-dependent visibility or surface selection. Across all three groups, viewpoint-dependent degradation remains largely unexplored.

**Table 8:** Viewpoint-related components modeled by representative attack methods. (✔) and (🞬) indicate the availability and unavailability of a component, respectively. A dash marks a field that could not be established from the record.

Method	Scale ↔ Altitude	Projection Geometry	Camera Pose Used	3D Surface Selection	Altitude Metadata	Angle Metadata	Viewpoint-Stratified Degradation	Physical Validation
AP-PA [4]	✔ (scale ratio)		🞬	🞬	🞬	🞬	🞬 (generic EOT)	✔
RSI MultiScale [5]	✔ (height-keyed)	🞬	🞬	🞬 (fixed roof placement)	✔ (capture height)	🞬	🞬	✔ (25–120 m)
URAdv [6]	✔ (nested patches)	🞬	🞬	🞬	🞬	🞬	Partial (weather, blur, and noise augmentation, but not viewpoint-conditioned)	🞬
CDUPatch [12]	✔ (multi-scale clipping)	🞬	🞬	🞬	🞬	🞬	🞬	🞬
Cui et al. [54]	✔ (through the projection matrix)	✔ (projection-based morphology adaptation)	Implicit, through the projection matrix	🞬 (roof quad fixed by manual annotation)	✔ (10–30 m distance bands, used to report results)	✔ (pitch bands 0–45°, used to report results)	🞬	✔ (printed patch on a scale vehicle model)
Digital-to-Physical [60]	🞬	🞬	🞬	On-object and contextual placements only	🞬	🞬	🞬	✔
Lu et al. [55]	✔ (per-object resizing)	🞬	🞬	🞬	🞬	🞬	🞬	🞬
EMA [58]	✔ (30% of the vehicle area)	🞬	🞬	🞬	🞬	🞬	🞬	✔ (printed patch on a scale car model)
PatchGen [67]	—	🞬	🞬	🞬	🞬	🞬	🞬	—
EVD4UAV [53]	✔ (patch resized per altitude band)	🞬	🞬	🞬 (fixed roof placement)	✔ (three bands, used to resize and to stratify)	🞬 (camera held near-vertical)	🞬 (altitude bands only, angle fixed)	🞬 (rendered benchmark)
FCA [7]	Full coverage	Via differentiable 3D rendering	✔ (renderer parameter)	Entire object surface (not angle-conditioned)	🞬	✔ (elevation swept 0–50°)	Partial (render-based transformations)	🞬
DE-DAC [44]	Full coverage	Via neural renderer	✔ (renderer parameter)	Entire object surface	🞬	✔ (rotation controlled)	Partial (render-based)	🞬
Geometry-aware target	✔	✔ (simulator ground truth)	✔	✔ (angle-conditioned)	✔	✔	✔	✔

### 5.4. Robustness, Cost, and Reproducibility

Most studies report physical robustness yet evaluate it using inconsistent protocols. Variations in viewpoint, weather, distance, illumination, and image quality are common, but the specific transformations vary substantially across papers. As a result, robustness claims are difficult to compare directly. Two observations nonetheless emerge. First, robustness is often achieved through increasingly broad augmentation strategies. Methods such as URAdv [6] and DTA [9] improve generalization by using a wide range of transformations, but this increases computational cost. Second, most of these transformations are viewpoint-agnostic, meaning that geometric effects are rarely modeled explicitly.

Reproducibility remains a significant concern. As summarized in Table 9, optimization schedules and hardware configurations are reported inconsistently, and they use high-performance hardware that may be costly and impractical for UAV deployment. Several studies further rely on proprietary datasets, custom simulators, or unpublished scene configurations. Consequently, exact replication is often difficult despite the precision with which attack effectiveness is reported.

### 5.5. Detector Architecture and Patch Attack Generalization

As shown in Table 7, the threat model is white-box in almost every case, although the transfer setting varies. Most attack methods use an untargeted objective and are evaluated primarily in digital settings, while only a limited number of studies use both digital and physical experimental setups. Of the 107 papers examined, 26 use at least one specific detector architecture. Within these 26 papers, YOLO variants [29] appear in 21, Faster R-CNN [27] in 10, SSD [28] in 3, and Mask R-CNN [1] in 1. Transformer-based or set-prediction detectors appear in 4 papers, including Swin [140], YOLOS [141], MVDeTr [142], and DETR [143]. No paper in the reviewed literature reports results on FCOS [30] or CornerNet [31], the two anchor-free detectors discussed in Section 3.1 that are particularly suited to small aerial targets.

We hypothesize that two factors produce this imbalance. First, almost every aerial attack optimizes an objectness loss. The YOLO series, up to YOLOv7, and other anchor-based detectors predict an objectness score at every candidate location, which gives such a loss a direct quantity to minimize. However, YOLOv8 removes this objectness score. Its detection head directly predicts class scores along with bounding-box coordinates, which are used for confidence filtering and non-maximum suppression (NMS). An attack on YOLOv8 therefore minimizes the highest class score at the target rather than a separate objectness score, which is what confidence suppression means for an anchor-free YOLO model. FCOS [30] and CornerNet [31] replace objectness with a centerness or keypoint response, and set-prediction detectors produce no comparable per-location objectness score at all. The second is the practical availability of YOLO, which is the detector most often retrained on UAV imagery and the one with the most accessible implementations.

The YOLO papers do not divide the corpus by head design. This is because, as mentioned previously, the YOLO family includes both anchor-based and anchor-free heads. Anchor-based variants use a separate objectness score alongside class probabilities, whereas anchor-free variants such as YOLOv8 use class scores without a separate objectness score. Specifically, despite variations in anchor usage, both YOLO approaches use dense prediction heads that produce predictions at locations on a regular image grid and report detections when their confidence scores exceed a given threshold. In both cases, an adversarial patch attack can suppress detection by driving the relevant confidence score at the target location below the threshold. This explains why a single study, such as CDUPatch [12], reports results on YOLOv3, YOLOv5, and YOLOv8 together, as shown in Table 7. The reported evidence therefore primarily reflects detectors with dense prediction heads. Moreover, literature coverage is weakest for set-prediction detectors, which appear in four papers, and is absent for FCOS and CornerNet. Conclusions about aerial patch attacks in this review therefore describe behavior on dense prediction detectors, and the objectness argument above applies only to the anchor-based ones among them. The same limitation applies to the defenses in Table 6, which are also evaluated almost entirely on the same detectors.

This imbalance in the evaluation of detector architectures in aerial-image-based adversarial patch research should not be interpreted as evidence that newer architectures are inherently safer. Work outside the aerial setting reports that adversarial examples do not transfer readily between convolutional networks and transformers in either direction [144], and that the robustness of a transformer varies with model size, training data, and the type of perturbation applied [145]. Transformers also degrade differently under occlusion and distribution shift, which follows from the way attention aggregates information across the whole image [146]. A perturbation aligned with the token grid remains effective against them, so a transformer is not robust to patch-shaped perturbations by construction [147]. These results come from image classification rather than aerial detection, so they mark an open question rather than a finding that carries over.

Two implications follow for this review. First, most studies evaluate attacks and defenses on one detector family and assume that the findings generalize to other architectures. However, a defense shown to be effective against a patch attack on YOLO may not provide the same protection for a set-prediction detector. Second, transformer-based detectors may respond differently to adversarial patches than convolutional detectors. Therefore, the limited coverage of different detector architectures in the reviewed literature remains an open research question.

## 6. Critical Discussion

This section first discusses the trade-offs in the performance of different attack configurations. It then discusses the limitations that recur across the reviewed works, along with the identified research gaps. Specifically, Table 10 outlines the identified research gaps.

**Table 10:** Research gaps by category, with the point in the literature where each becomes visible.

Category	Gap	Evidence in the Literature
Technical	Viewing angle is handled primarily as scale variation; no angle-conditioned surface selection.	AP-PA [4], RSI MultiScale [5], URAdv [6], and CDUPatch [12] all resize the patch. Only Cui et al. [54] considers projection geometry, while Digital-to-Physical [60] compares an on-object placement with a contextual one rather than selecting a surface from the viewing geometry.
Technical	Physical degradation is sampled from a generic distribution rather than stratified by viewpoint.	Generic EOT augmentation is used throughout the aerial attack literature [4,5,6].
Technical	Naturalness priors are not conditioned on altitude or sensor characteristics.	EMA [58] reports that ground-oriented naturalness does not transfer well to UAV imagery.
Evaluation	Attacks are frequently evaluated only on training-detector weights, with sparse physical experiments and limited ablation studies.	Section 6.4.
Dataset	The real UAV datasets this literature evaluates on carry no camera intrinsics or pose, whereas simulators provide geometry but introduce sim-to-real uncertainty. Where coarse altitude is logged and used, as in EVD4UAV [53], it selects a scale factor and a results split rather than entering a geometric model.	Section 3.4 and Table 3.
Dataset	Aerial infrared and multi-modal datasets remain limited.	MSDrone [12] was introduced to address this gap.
Evaluation	Segmentation models are largely absent from the aerial attack and defense literature, although they are deployed for the same identification task.	Patch attacks on segmentation are evaluated on driving imagery [13]. No aerial counterpart was returned by the detection-scoped search of Section 2, which is a weaker basis than the other rows and is flagged as such in Table 11.
Evaluation	No common aerial benchmark exists; evaluation metrics are difficult to compare, and black-box, adaptive, and transformer-based detectors remain under-tested.	Section 5.1; [101,102,137].
Defense	Defenses are designed and tested against planar patches seen from a fixed viewpoint. We did not identify one that conditions its response on altitude or look-down angle, or one evaluated against camouflage or non-contact perturbations.	Section 4.6; Pathak et al. [14] is the only UAV-specific defense in the corpus.
Defense	The on-board cost of a defense, meaning inference overhead, memory, and behavior after quantization, is rarely reported alongside robust accuracy.	Masking-based certification requires repeated inference on masked copies of the image [65,66]; PatchBlock [99] is the one defense written for embedded hardware; quantization is examined only in [100].
Deployment	Attack generation and detector inference under the latency and power budgets of an aerial platform remain underexplored.	Section 6.6.

### 6.1. Trade-Offs in Attack Design and Effectiveness

Some patch attacks target the objectness score of the detector, whereas others manipulate the class score to influence the detection outcome. Based on the reviewed studies, most of the works adopt the former approach.

Objectness determines whether a candidate box is considered to contain an object, so suppressing it can directly eliminate the corresponding detection. In contrast, a class-manipulation attack typically acts on a box that remains detected and primarily changes the class assigned to that box. In one-stage anchor-based detectors, including the YOLO releases up to YOLOv7, objectness and class scores are predicted together and contribute to the confidence used for filtering detections. Therefore, strongly suppressing the objectness score can reduce the final detection confidence regardless of the predicted class. In two-stage detectors, the distinction is also reflected in the architecture, as a region that is not generated by the region proposal network is not passed to the subsequent detection head [4]. Figure 9 illustrates these two attack strategies.

The frequent use of objectness losses in the reviewed studies reflects the preference for this approach in the field. This is mainly due to the structure of the evaluated detectors, for which objectness suppression provides a more direct way to remove a detection. However, this does not apply equally to all detector architectures. Anchor-free detectors such as FCOS [30] and CornerNet [31] use centerness or keypoint heatmaps instead of objectness, while end-to-end set-prediction detectors do not use the same type of detection gate. Anchor-free YOLO variants, such as YOLOv8, also belong to this group. However, they use a class score predicted by the detection head at the target location, which an attacker can suppress in the same way as the objectness score in anchor-based variants. Figure 9 illustrates these differences. Whether objectness-style suppression has a direct equivalent in these detector families remains an open question, and Section 5.5 discusses the limited coverage of these architectures in the reviewed studies.

A similar trade-off can be observed when comparing a planar patch with full-surface camouflage. Under substantial changes in viewpoint, a planar patch may become foreshortened or move outside the visible silhouette of the target, whereas a texture applied across the object’s surface can continue to present adversarial content to the camera [7]. However, this increased viewpoint robustness requires a 3D model of the object, a differentiable renderer, and evaluation within a simulated environment. In contrast, planar patch approaches require a simpler attack pipeline and can be evaluated on real-world imagery. Therefore, the preferred approach depends on the degree of viewpoint variation expected in the deployment environment.

Transferability of the attacks presents a similar trade-off: self-ensemble training can improve transferability across different detector architectures by reducing dependence on a single model [103], whereas a patch optimized for a specific known detector may achieve higher effectiveness against that detector. Overall, attacks tend to perform better when their formulation matches the structure of the target system, such as its detection outputs, object geometry, or detector architecture. Multi-view detection is a case in point. Fool the Hydra [62] reports that patches built for a single view leave MVDet [148] and MVDeTr [142] largely intact, even when every person in every view carries one, because the two detectors combine evidence across cameras. Attacks that use the same cross-view information are effective. A patch optimized with gradients aggregated across views lowers the MODA of MVDet by 73.77%, and a separate patch that also suppresses the attention of MVDeTr lowers its MODA by 62.57%. The MVDet patch does not transfer to MVDeTr, so the result rests on two attacks rather than one.

Finally, the naturalness of patch attacks presents another trade-off with attack performance. EMA reports a loss of at most two points in mAP when the patch is designed to match its environment [58], while other studies report larger performance losses.

### 6.2. Hidden Assumptions

Several assumptions recur throughout the aerial adversarial-attack literature but are rarely stated explicitly.

The first assumption is that the UAV observes the scene from a near-nadir viewpoint. This assumption is deeply embedded in both benchmark datasets and experimental demonstrations. It also explains why patch resizing has proven so effective. Under near-nadir viewing, changes in altitude primarily affect the apparent size of the target, making scale adaptation a reasonable approximation of viewpoint variation.

Scale is the dominant first-order effect of altitude, but it does not constitute a complete physical model of altitude variation. Increasing altitude can lengthen the atmospheric path, which may reduce image contrast and increase haze. It also changes the target’s pixel size relative to the detector’s receptive field and anchor configuration, and can interact with motion blur. These effects can influence whether an adversarial perturbation remains effective. Therefore, a patch may fail even when its scale is adjusted correctly if the target features become too small to be resolved in the image. Hence, it is reasonable to argue that scale is the main factor considered when evaluating the effect of altitude, but other factors that are not explicitly modeled may also affect attack performance.

As mentioned previously, this assumption appears in most of the reviewed studies, where patch size is coupled to altitude, placement is fixed to a predetermined region of the target, and viewpoint variation is represented primarily through generic transformation augmentation. However, this assumption may fail under different viewing and environmental conditions (e.g., rain and fog), as the visible surfaces of the object vary with the viewpoint, while adverse environmental conditions can affect image quality and visibility. Figure 10 illustrates this distinction in viewpoint variation.

The second assumption is white-box access to the object detector. Although black-box and adaptive-detector settings have received increasing attention [101,102], most reported studies still develop their attacks under the assumption of white-box access to the network.

### 6.3. Reproducibility

Although performance results are reported in detail, the information needed to reproduce those results is often incomplete. ASRs are often reported with high precision, while important details such as optimization schedules, hyperparameters, patch resolution, and hardware configurations are often incomplete or omitted. Several studies also depend on private datasets and custom simulators that are not publicly released. Reproducing the reported results is therefore difficult without access to the original assets. Recent efforts to re-evaluate defenses under common experimental protocols [137] represent an important step toward greater reproducibility.

### 6.4. Gaps in Data, Geometry, and Evaluation

The limitations above leave gaps that recur across the reviewed work. Table 10 lists them by category together with the point in the literature where each becomes visible. The reproducibility row records what Section 6.3 has already argued and is not restated here. This subsection covers the technical, dataset, and evaluation gaps, and Section 6.5 covers the defense-side ones.

The technical gaps share one root. Because aerial methods model viewpoint through object scale, angle-conditioned surface selection, viewpoint-specific degradation modeling, and altitude-aware notions of naturalness are absent from most existing approaches. Their absence reflects the limited geometric information available in the datasets used for evaluation rather than any lack of methodological sophistication, and that dataset limitation underlies most of the remaining gaps. The real aerial benchmarks most commonly used in adversarial attack research do not provide the camera intrinsics, pose information, or scene geometry needed to study viewpoint systematically. Conversely, simulation environments provide precise geometric information but introduce uncertainty regarding real-world transferability. Datasets such as EVD4UAV [53] and HIT–UAV [52] include coarse altitude or viewpoint information. The authors of EVD4UAV use it to stratify the benchmark and to resize the patch. Beyond that, we did not identify further use of this metadata within the corpus, and in no case did we identify it being used as a camera parameter in the sense set out in Section 3.4. As a result, current research is forced to choose between realistic imagery with limited geometric information and geometric fidelity with limited real-world validation.

This challenge is further compounded by inconsistent evaluations. Existing studies employ different datasets, detector architectures, attack sizes, and evaluation metrics, making direct comparison difficult. Robustness assessments are typically conducted over a limited set of viewpoints and environmental conditions, while challenging settings such as black-box attacks, adaptive defenders, transformer-based detectors, and continuous viewpoint variation receive comparatively little attention. Without a common aerial benchmark that standardizes evaluation while preserving viewpoint diversity, progress remains difficult to measure consistently across studies.

Three evaluation practices weaken the evidence further. Physical trials are usually small and are run at a few hand-picked angles and distances rather than sampled across the altitude and viewpoint range a UAV covers. Attacks are often measured on the same detector weights used to optimize them, which shows that training succeeded but says little about an unknown model. Ablations are uncommon, so a paper that changes the loss, the transformation set, and the placement rule at once does not separate the contribution of each.

Seen from a distance, the field is mature in attack optimization, physical realization, and transfer, and much less mature in how it represents viewpoint. Objectness losses, scale adaptation, and steadily broader augmentation are settled practice. Claims about viewpoint robustness, though, tend to rest on augmentation and spot checks rather than on geometry anyone measured, which is unsurprising given that the real aerial benchmarks in common use record none.

### 6.5. Gaps in Adversarial Defense Research

The adversarial patch defense literature also has limitations, particularly in the context of aerial object detection.

The first limitation is that most defenses are designed and evaluated against planar patches viewed from a fixed viewpoint. As discussed in Section 4.6, most of the reviewed defenses focus on detecting, masking, or filtering a compact printed region in ground-level images, without considering factors such as altitude or viewing angle. These factors are important in aerial settings because they affect both the apparent size of the target and the visible surfaces on which a patch can be placed. We also did not identify any reviewed defense method evaluated against full-surface camouflage or non-contact perturbations, such as projected light patterns.

The second limitation is computational cost. Most studies report robust accuracy but do not report important deployment factors such as throughput, memory usage, or power consumption. For example, masking-based certification requires the detector to process multiple masked versions of the same image [65,66], while restoration-based defenses using diffusion models add a generative step to each frame [129]. However, the computational cost of these methods on UAV hardware is rarely evaluated. Quantization, which is commonly applied when deploying models on embedded devices, has also received very limited attention in adversarial patch defense research [100]. Therefore, the most studied defenses are not necessarily the most practical for UAV deployment.

### 6.6. Practical and Deployment Challenges

Developing patch attacks in digital simulations and evaluating them in the real world introduces several challenges. Factors such as rendering fidelity, material properties, sensor noise, adverse environmental conditions such as weather, and printing distortions can influence physical attack performance, but accurately modeling and systematically evaluating these factors remains challenging.

Second, computational cost applies to some parts of this problem and not to others. A printed patch is produced before the flight, so the attacker pays nothing at inference time and the constraint does not bind. It binds on the defense, which runs on the aircraft inside the frame budget of the detector, and on scene-specific patch generation, which would have to run while the target is being observed. UAV platforms operate under strict power, memory, and latency limits, so both cases have to be light enough for embedded hardware. A similar challenge has been identified in connected and autonomous vehicles, where adversarial machine learning must operate within the vehicle’s real-time latency and safety constraints rather than being handled offline [24].

Third, conditioning a patch on viewpoint geometry in real time is difficult, although this review argues that it matters. The attacker would need the viewing geometry of a UAV it does not control. That could come from onboard telemetry, from signals between the aircraft and its controller, from V2X messages, or from visual estimation, and each route carries latency and localization error. A printed patch also cannot be repositioned once it is fixed, so an angle-dependent placement rule would need a non-contact carrier such as a pattern projected from a second aircraft. Section 4.4 sets out why projection from an aerial standoff is itself unproven.

Finally, conducting large-scale aerial experiments when developing attack or defense methods introduces practical costs, airspace regulations, safety requirements, and broader dual-use considerations.

Table 11 summarizes the limitations identified in this section together with the evidence behind each one.

**Table 11:** The limitations identified in this review, with the evidence behind each one.

Limitation	Claim or Evidence from the Reviewed Literature
Viewing angle is handled predominantly through scale adaptation rather than through explicit geometry.	Checked across the corpus. A single aerial attack that makes surface choice or projection a function of measured viewing geometry would overturn it.
No attack in the corpus selects the target surface as a function of look-down angle.	Established at method level. Cui et al. [54] come closest, warping the patch onto a roof quad whose corners are annotated by hand, so the carrier surface is fixed before optimization. A method whose placement rule reads a camera parameter would overturn it, and the property is visible from a method description, so a missed instance is unlikely.
Physical degradation is sampled from a generic distribution rather than stratified by viewpoint.	Established at method level. An augmentation schedule conditioned on elevation angle would overturn it. This turns on implementation detail and is the claim most exposed to a missed case.
Recorded altitude metadata is used to stratify results and to resize the patch, not as a camera parameter.	Checked across the datasets in Table 3. Cui et al. [54] report results by distance and pitch band without feeding either into the attack. A study consuming EVD4UAV or HIT–UAV metadata to reason about projection or visibility would overturn it.
No camouflage study reports results at near-nadir elevation.	Established at method level. FCA [7] sweeps elevation from 0 to 50 degrees, which is the widest range identified. A sweep reaching beyond roughly 60 degrees would overturn it.
No defense conditions its response on altitude or look-down angle, and none was evaluated against camouflage or non-contact perturbations.	Checked across the defenses in Table 6. A defense reporting robustness as a function of viewing geometry, or evaluated against a full-surface texture, would overturn it.
On-board cost is rarely reported alongside robust accuracy.	Checked across the corpus. Three sources report a cost figure. DetectorGuard [64] gives a per-module runtime breakdown, NutNet [139] gives an inference-time increase, and PatchBlock [99] targets embedded hardware. None of the three is aerial, so the claim is one of rarity rather than absence. Throughput and memory reported on UAV-class hardware would overturn it.
Aerial segmentation attacks are absent from the literature.	Limited by what was searched for. The search was scoped to detection, so this records what was not encountered rather than what does not exist, and it is reported as a research direction rather than a finding.
No prior review organizes its analysis around aerial viewpoint geometry.	Checked across the surveys in Table 1. Chen and Chu [25] review aerial adversarial robustness but organize it by sensing modality and operational phase. A survey with an altitude-by-angle organizing structure would overturn it.
Objectness suppression outperforms class manipulation.	Not established, and argued structurally rather than measured. Section 6.1 gives the architectural reason and notes that no controlled comparison in the corpus measures the difference.

## 7. Future Directions

The gaps identified in the previous section mainly highlight that future research should treat viewpoint geometry as a fundamental component of aerial adversarial learning, rather than as a nuisance variable addressed solely through generic augmentation. Figure 11 summarizes five future directions that emerge from this observation and maps them onto the corresponding gaps in the literature.

1. **Geometry-aware attacks**: Existing aerial methods primarily adapt patch size to altitude while keeping placement fixed. Future work could explicitly model placement, visibility, and degradation as functions of viewing geometry. Simulators provide a practical environment for investigating such ideas because they offer exact pose, depth, and surface information. A UAV simulator such as AirSim [49] is a promising platform for this, because camera intrinsics, altitude, and pose are available through its programming interface and can be varied systematically. Existing studies provide partial evidence that geometric reasoning can be beneficial, including Cui et al.’s [54] projection-transformation attack and the comparison of on-object and contextual placement reported by study [60]. Extending these concepts toward viewpoint-aware attack formulations represents a logical extension of current research. Two practical constraints apply to any such attack. The attacker must recover the viewing geometry of a UAV that is not under their control, and a printed patch cannot be repositioned once it is fixed, so a placement rule that depends on the current angle would need a non-contact approach such as a projected pattern. Section 6.6 sets out both constraints.
2. **Simulation-to-reality transfer**: Viewpoint geometry is easiest to study in simulation, where scene structure and camera pose are known precisely, yet practical relevance ultimately depends on performance in real aerial environments. Future studies could therefore treat simulation and real-world evaluation as complementary sources of evidence, using simulation for controlled geometric analysis and real imagery for validating transferability. Study Digital-to-Physical [60] illustrates why this matters, because the placement that performed best in its digital evaluation was not the one that performed best after physical deployment. Such an approach would make simulation-to-reality transfer an explicit and measurable aspect of evaluation rather than an implicit assumption.
3. **Naturalness and performance under domain shift**: Existing appearance priors are largely derived from ground-level imagery and may not reflect how objects appear from UAV altitudes or under alternative sensing modalities. EMA’s findings [58] suggest that appearance constraints optimized for ground-level imagery do not necessarily transfer to aerial environments. Future research may therefore benefit from altitude-aware and sensor-aware appearance models derived directly from aerial imagery. Further, adverse weather conditions, such as fog, which can significantly affect the performance of adversarial patch attacks, should also be investigated.
4. **Viewpoint-aware and deployment-oriented defenses**: Future research should develop adversarial patch defenses that explicitly incorporate UAV viewpoint information, such as altitude and camera angle, rather than relying solely on generic augmentation. These defenses should also be evaluated against diverse attack types, including camouflage and non-contact perturbations, and tested on embedded UAV hardware. Similar research is needed for other aerial perception tasks, such as segmentation and depth estimation, which remain largely unexplored in the reviewed literature.
5. **Standardized aerial benchmarking platform**: Although EVD4UAV [53] demonstrates altitude-aware evaluation and HIT–UAV [52] demonstrates the value of viewpoint stratification, we did not identify a benchmark that combines realistic aerial imagery, viewpoint information, standardized detectors, and consistent evaluation protocols within a single framework. An advanced simulation environment incorporating such a standardized aerial benchmark would be beneficial for controlling geometric factors and environmental conditions, as well as evaluating the performance of attack and defense methods over long-term video sequences.

## 8. Conclusions

Adversarial patch and camouflage attacks against aerial object detection have advanced rapidly over the past decade. The literature has converged on effective objectness-suppression objectives, increasingly robust physical attack formulations, and sophisticated approaches to transferability and camouflage optimization. At the same time, the review reveals a recurring limitation. Viewpoint variation in aerial imagery is typically represented through changes in scale, while the broader geometric consequences of viewing angle receive comparatively little attention.

A central finding of this review is that this limitation is largely due to how existing studies model and evaluate viewpoint variation. The benchmark datasets most commonly used for aerial attack research provide realistic imagery but limited geometric information, whereas simulation environments provide exact geometry but limited evidence of real-world validity. Even when flight metadata is available, it is mainly used to stratify results or select patch scale rather than to explicitly model the geometric effects of viewpoint variation. We did not identify any study in the reviewed corpus that explicitly incorporates viewpoint information as a camera parameter to govern surface selection and attack projection.

Progress therefore depends on how viewpoint geometry is represented, and not on attack or defense strength alone. Three directions follow from this. Patch naturalness should be studied on aerial imagery rather than borrowed from ground-level priors. Attack and defense performance should be measured under the domain shifts and adverse weather that aerial platforms meet. Defenses should be designed to run within the memory and latency budget of embedded UAV hardware.

## Figures and Tables

**Figure 1:**
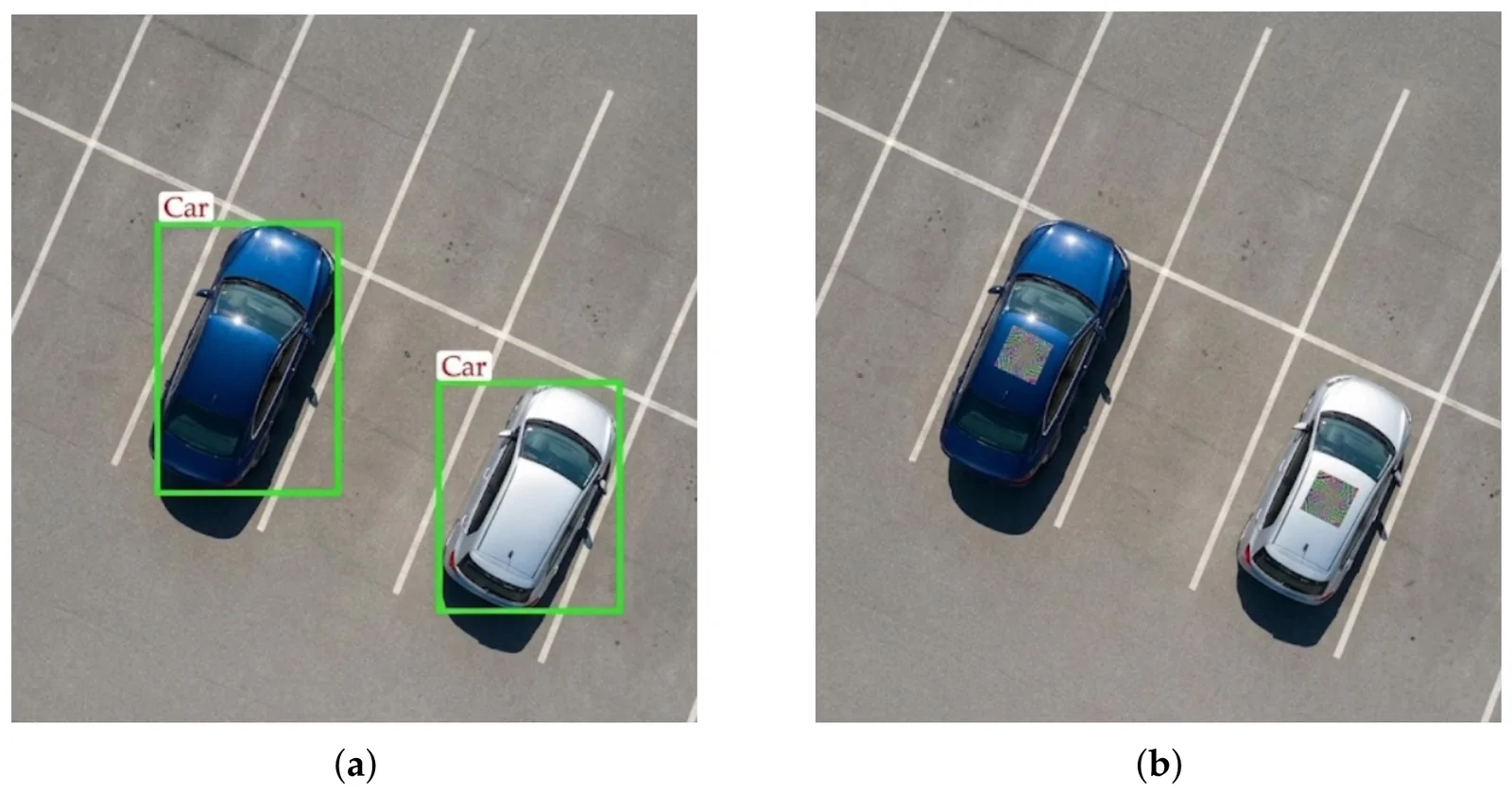
Conceptual illustration of an adversarial patch attack against an aerial object detector. (**a**) The detector identifies the vehicles. (**b**) The same vehicles are missed once a patch is placed on them. The figure is illustrative and shows no measured detector output or experimental result.

**Figure 2:**
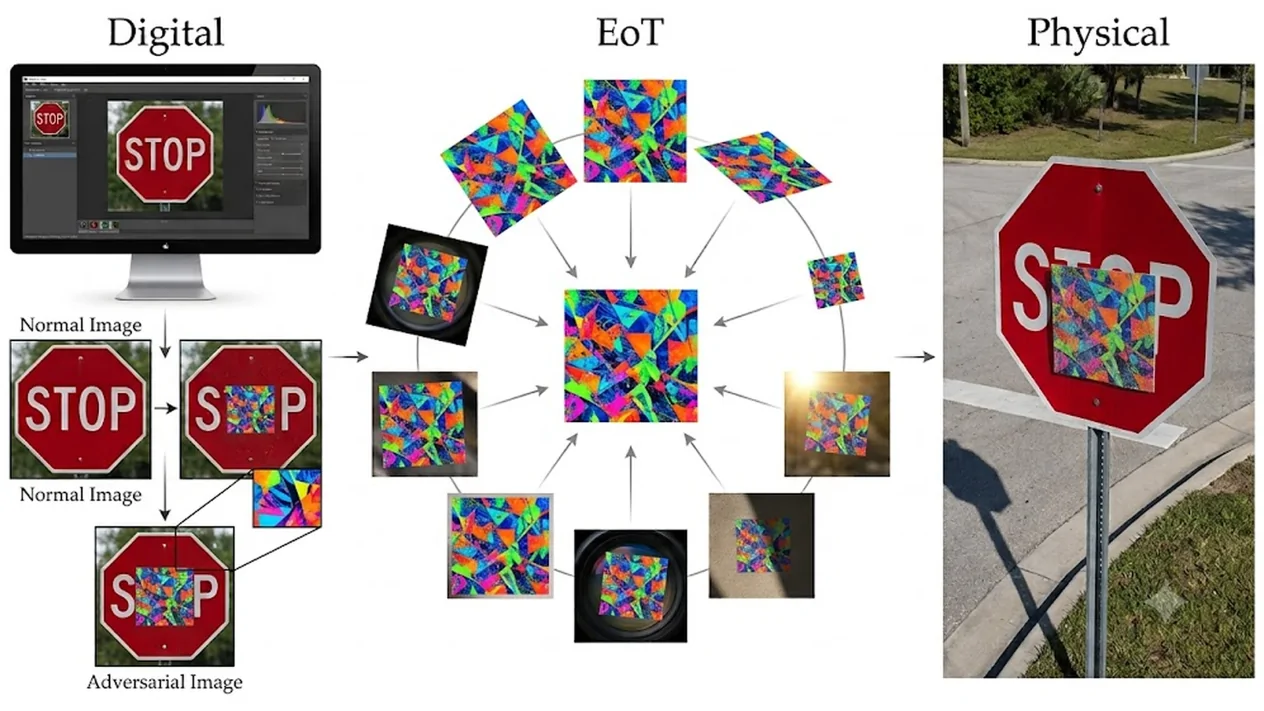
Conceptual illustration of an adversarial patch attack in digital and physical environments. The figure is illustrative and shows no measured detector output or experimental result.

**Figure 3:**
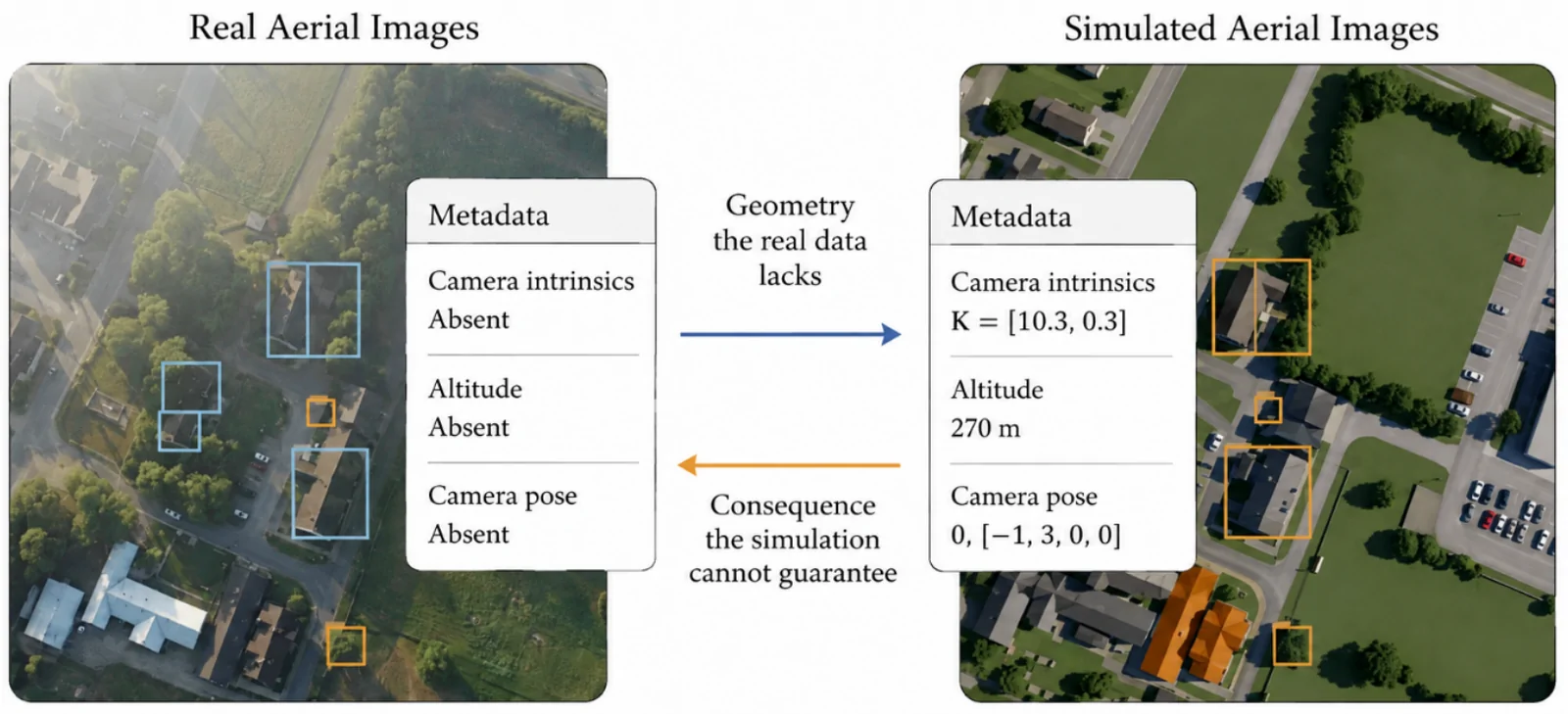
Conceptual illustration of the data paradox: real aerial benchmarks record detections but not viewing geometry, whereas renderers supply exact camera parameters. The figure is illustrative and shows no measured detector output or experimental result.

**Figure 4:**
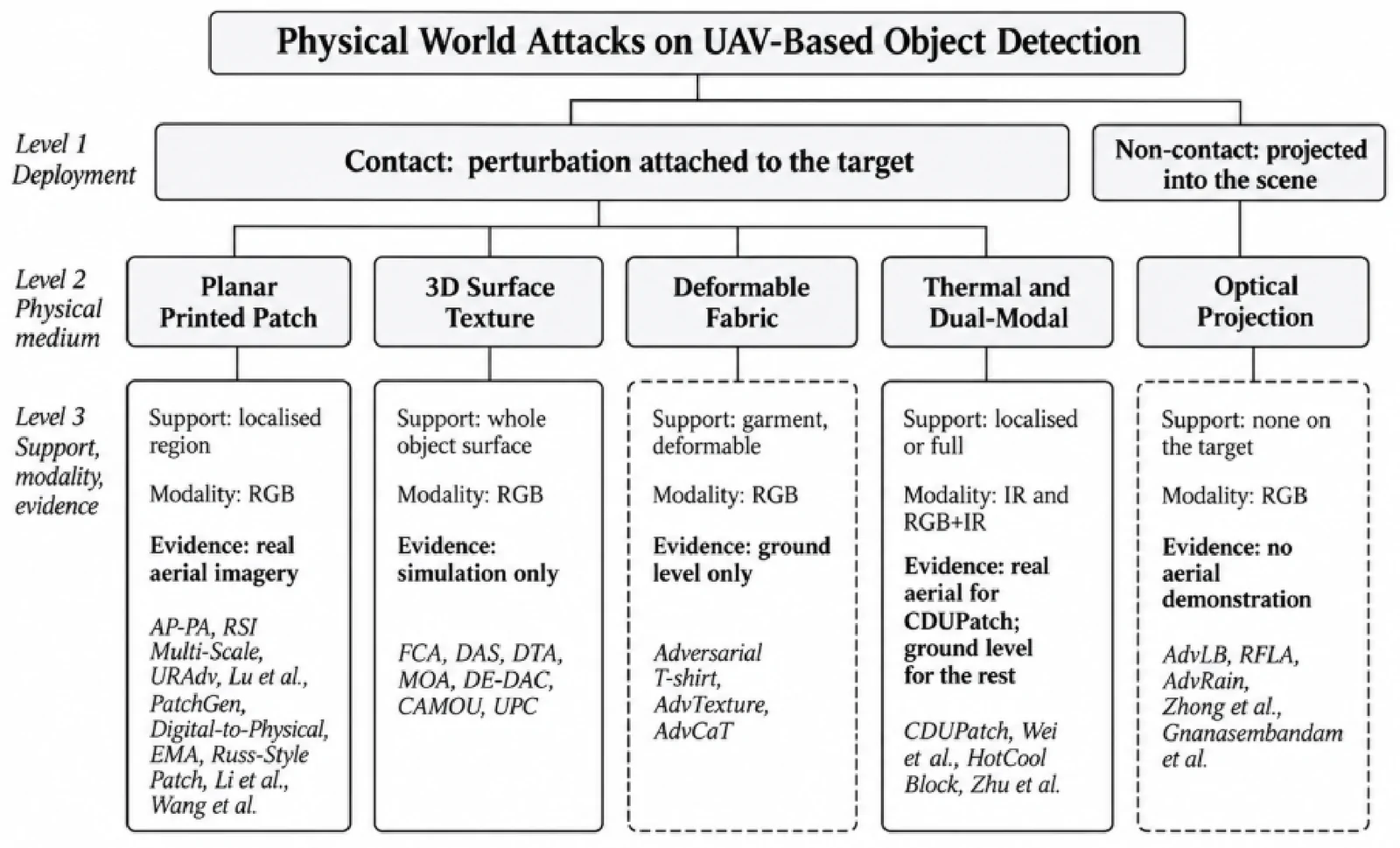
A taxonomy of physical-world attacks on UAV-based object detection, with deployment, physical medium, and per-medium properties held on separate levels. A dashed border marks a carrier for which no aerial evaluation is reported. The methods named in the figure are as follows. Planar printed patch: AP-PA [4], RSI Multi-Scale [5], URAdv [6], Lu et al. [55], PatchGen [67], Digital-to-Physical [60], EMA [58], Rust-Style Patch [68], Li et al. [69], and Wang et al. [70]. 3D surface texture: FCA [7], DAS [8], DTA [9], MOA [63], DE-DAC [44], CAMOU [71], and UPC [72]. Deformable fabric: Adversarial T-shirt [73], AdvTexture [74], and AdvCaT [75]. Thermal and dual-modal: CDUPatch [12], Wei et al. [10], HotCold Block [76], and Zhu et al. [11]. Optical projection: AdvLB [77], RFLA [78], AdvRain [79], Zhong et al. [80], and Gnanasambandam et al. [81].

**Figure 5:**
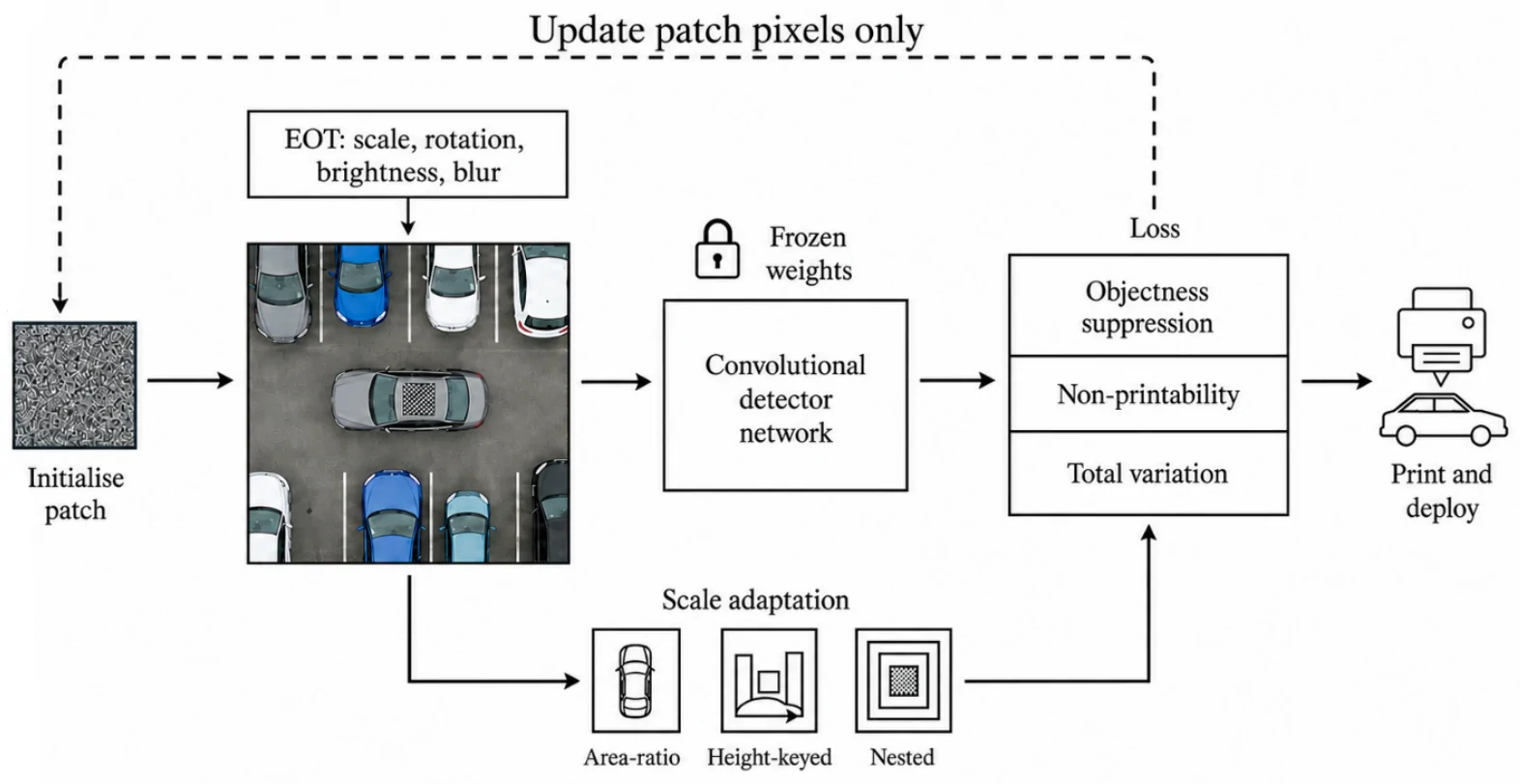
Conceptual illustration of the shared aerial patch pipeline, in which a patch is composited under EoT and optimized through a frozen detector.

**Figure 6:**
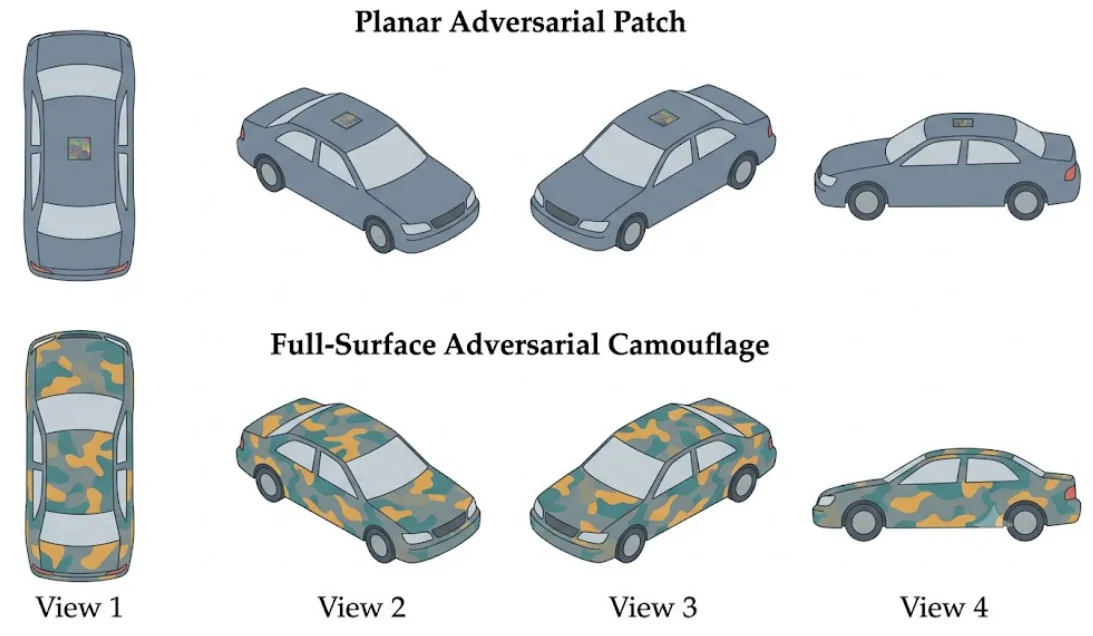
Conceptual illustration showing that a planar patch may become less visible or leave the silhouette under rotation, while full-surface camouflage remains visible. The figure is illustrative and is not derived from experimental results.

**Figure 7:**
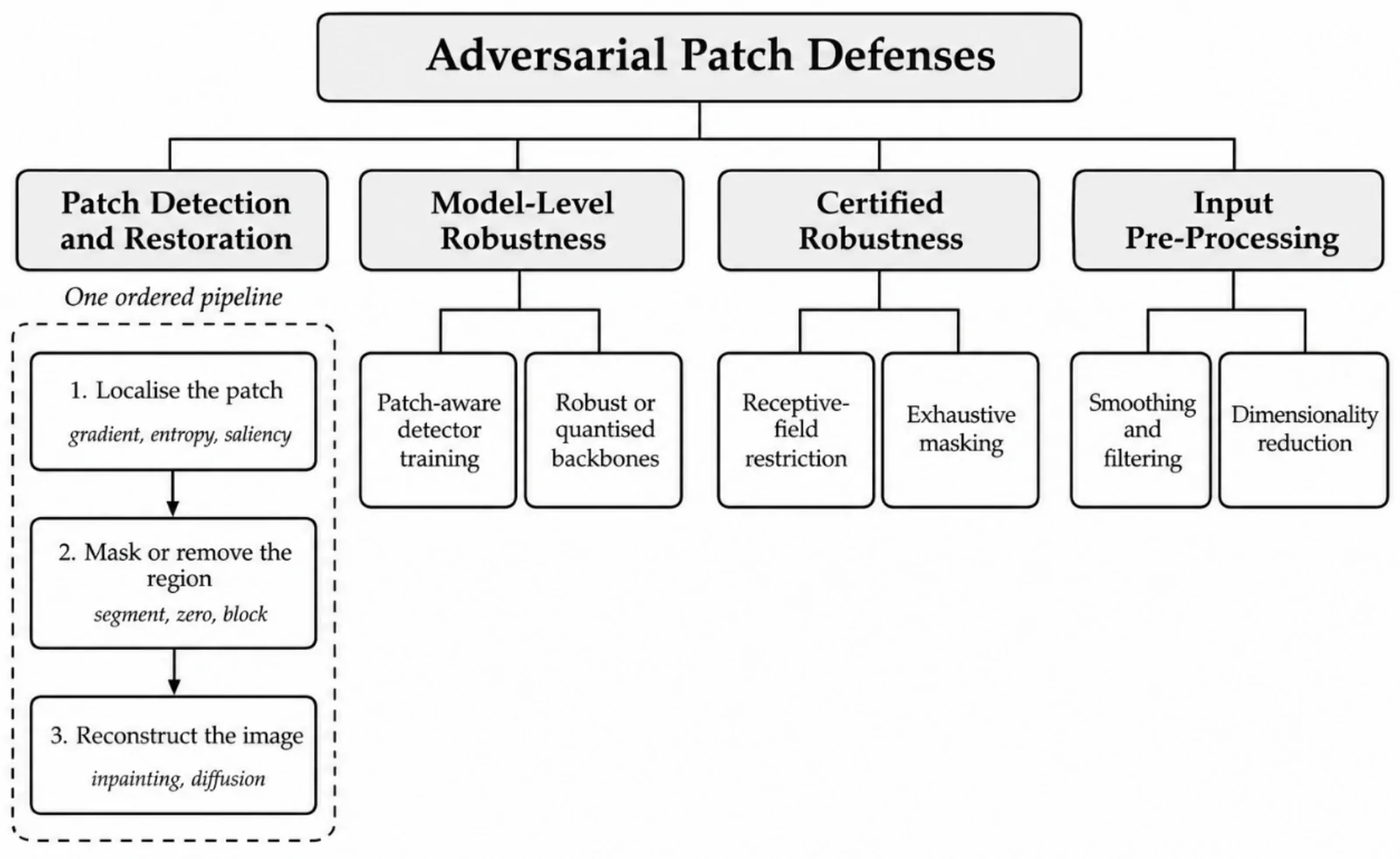
The four families of adversarial patch defense methods.

**Figure 8:**
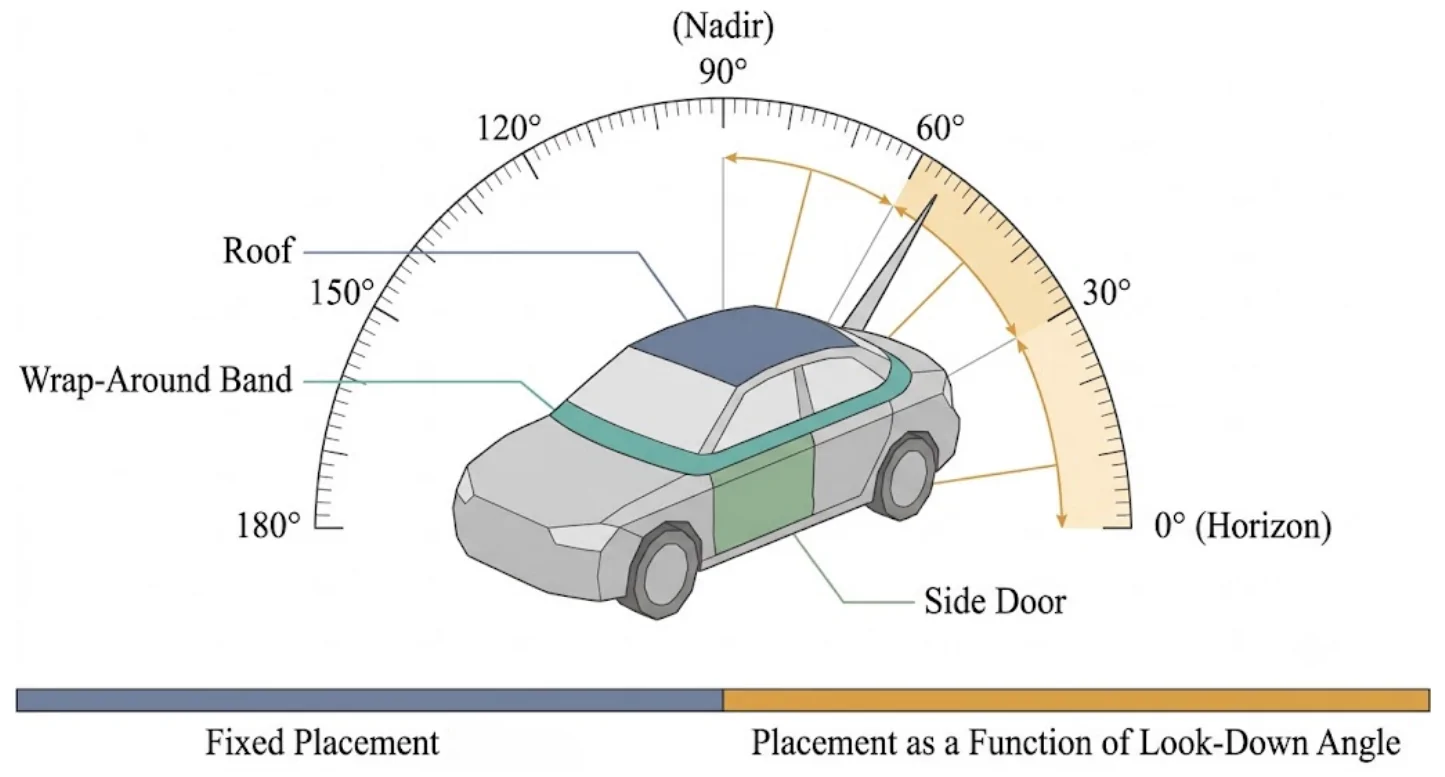
Conceptual illustration of the angle-conditioned placement. Rather than relying on fixed or manually specified patch locations, placement is conditioned continuously on the look-down angle and the surface visible to the camera.

**Figure 9:**
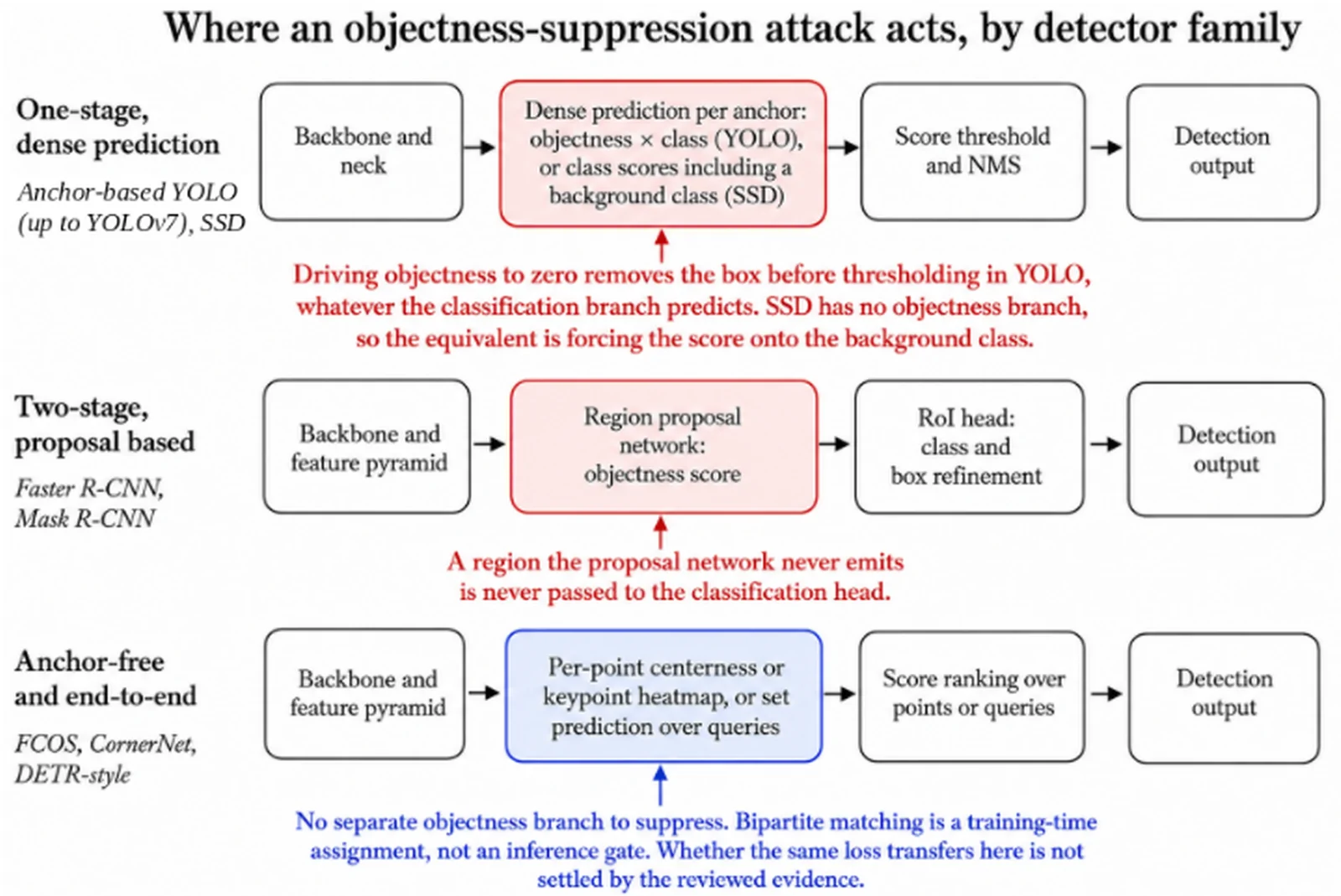
Schematic illustration of the objectness-suppression attack across three detector families: one-stage anchor-based, two-stage anchor-based, and anchor-free or set-prediction detectors.

**Figure 10:**
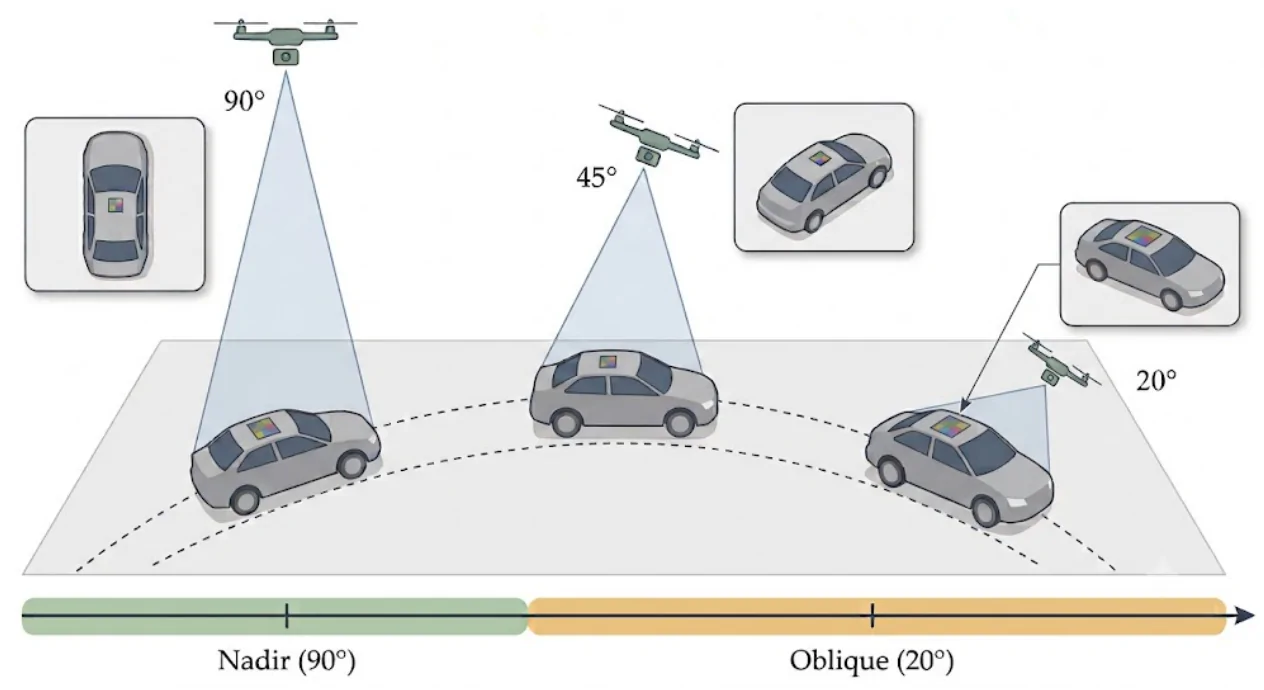
Conceptual illustration of how the imaging condition changes with look-down angle. Near-nadir imagery is dominated by scale, whereas lower look-down angles also change the visible surface and the image projection.

**Figure 11:**
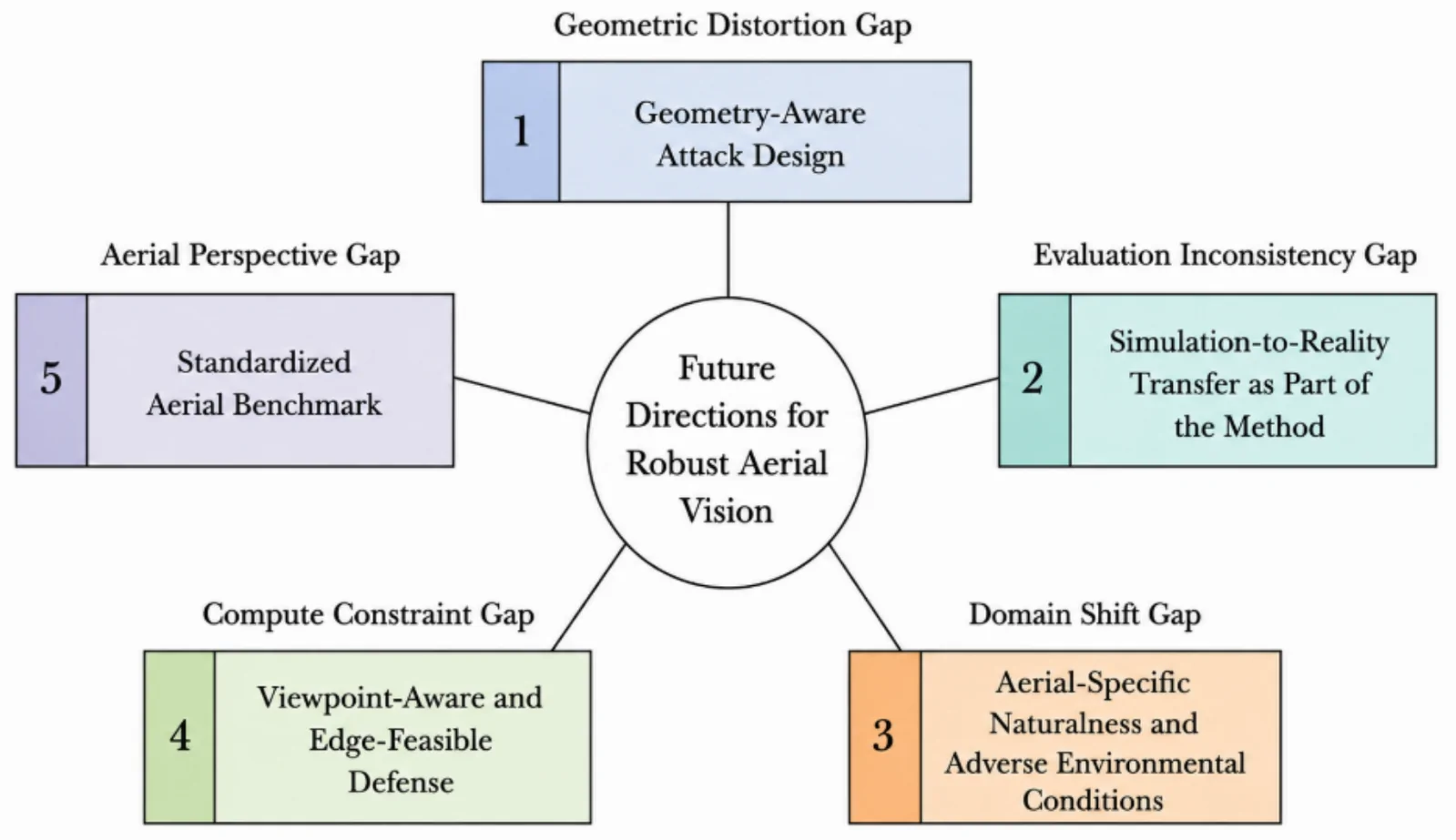
Five future research directions and the constraints on them, covering sim-to-real transfer, adverse environmental conditions, on-board computation, a standardized aerial benchmark, and field deployment.

**Table 1:** Comparison of this review with existing adversarial-attack surveys. • = covered in depth; •∘ = partially covered; ∘ = not covered.

Survey	Year	Physical Patches	Aerial/UAV Focus	3D Camouflage	Viewpoint Geometry
Wang et al. [18]	2022	•	∘	•∘	∘
Wei et al. [20]	2024	•	∘	•∘	∘
Guesmi et al. [17]	2023	•	•∘	∘	∘
Amirkhani et al. [21]	2023	•∘	∘	∘	∘
Nguyen et al. [22]	2025	•∘	∘	∘	∘
Wang et al. [23]	2023	•	∘	•∘	∘
Chen & Chu [25]	2024	•	•	∘	∘
Liu & Xu [19]	2025	•	∘	∘	∘
This Review	2026	•	•	•	•

**Table 2:** Distribution of the 107 retained papers across thematic clusters.

Thematic Cluster	Papers	Thematic Cluster	Papers
Defense	24	Loss/generation technique	7
UAV/aerial attack	17	Naturalistic/stealth attack	5
3D/multi-view camouflage	11	Multi-view systems and 3D detection	4
Transferability	9	Person/wearable attack	3
Foundational/baseline	8	Survey/reference	3
Optical/non-contact attack	8	Adjacent (face/person)	1
Infrared/multi-modal attack	7	**Total**	**107**

**Table 4:** Evaluation metrics used across the reviewed literature. TP, FP and FN denote true positives, false positives and false negatives, p(r) the precision at recall *r*, *C* the number of classes, and $N_{\mathrm{GT}}$ the number of ground-truth objects.

Metric	What It Measures	Definition	Preferred Direction
AP/mAP (@IoU)	Detection quality, measured as the area under the precision–recall curve at a specified IoU threshold.	$\mathrm{AP}=\int_{0}^{1}p\left(r\right)\;dr$, $\mathrm{mAP}=\frac{1}{C}\sum_{c=1}^{C}{\mathrm{AP}}_{c}$	Higher is better for the detector, so a larger drop indicates a stronger attack.
ASR	Attack success rate, i.e., the fraction of targets successfully suppressed or misclassified.	$\mathrm{ASR}=N_{\mathrm{success}}/N_{\mathrm{total}}$	Higher indicates a stronger attack. The definition of success varies across studies.
Precision/Recall	False-positive and false-negative behavior of the detector.	$P=\frac{\mathrm{TP}}{\mathrm{TP}+\mathrm{FP}}$, $R=\frac{\mathrm{TP}}{\mathrm{TP}+\mathrm{FN}}$	Higher is better for the detector.
MODA	Multi-object detection accuracy, used primarily for multi-view detection benchmarks, e.g., Fool the Hydra [62].	$\mathrm{MODA}=1-\frac{\mathrm{FP}+\mathrm{FN}}{N_{\mathrm{GT}}}$	Higher is better for the detector.
Robust AP/mAP	Detection quality retained by a defended model when the input carries an adversarial patch.	AP or mAP of Equation above, computed on attacked inputs	Higher is better for the defense. Reported next to the clean value to expose the cost of the defense.
Certified accuracy	Proportion of inputs for which a certified defense guarantees a correct outcome against any perturbation inside its threat model.	Guarantee derived from the certification procedure, not from a closed-form expression	Higher is better for the defense. It is a lower bound, so the empirical value is never worse.

**Table 9:** Implementation details reported by the attack methods most relevant to this review. None report a training cost in wall-clock or GPU-hours.

Method	Optimizer/Schedule	Hardware
AP-PA [4]	Adam, 600 epochs	RTX 3080
RSI Multi-Scale [5]	Loss maximization, 200 epochs, batch size 3	RTX 3090
URAdv [6]	Adam-AMSGrad, learning rate 0.03, 400 epochs	RTX 3090
PatchGen [67]	Adam, 300 epochs, learning rate 0.01	RTX 3090
FCA [7]	Adam (differentiable renderer)	RTX 3090

## Data Availability

The per-paper corpus underpinning this review, together with the fields extracted from each paper, is available at https://github.com/sandeshshrestha45/Adversarial-Patch-and-Camouflage-Attacks-on-Aerial-Object-Detection (accessed on 17 September 2026).

## Abbreviations

The following abbreviations are used in this manuscript.

AdvART	Adversarial Art	Jedi	Entropy Based Patch Defense
AdvCaT	Adversarial Camouflage Texture	LLVIP	Low-Light-Visible Infrared Paired Dataset
AdvLB	Adversarial Laser Beam	mAP	Mean Average Precision
AdvRain	Adversarial Rain	Mask R-CNN	Mask Region Based Convolutional Neural Network
AdvTexture	Adversarial Texture	MeshAdv	Mesh-based Adversarial Attack
AirSim	Aerial Informatics and Robotics Simulation	MOA	Mimic Octopus Attack
AP	Average Precision	MODA	Multi-Object Detection Accuracy
AP-PA	Adaptive Patch-Based Physical Attack	MSDrone	Multi-Spectral Drone Dataset
BB	Black-box (attack setting)	MVDeTr	Multi View Detection Transformer
CAMOU	Learned Vehicle Camouflage Attack	MVDET	Multi-View Detector
CARLA	Car Learning to Act (driving simulator)	NPS	Non-Printability Score
CARPK	Car Parking Lot Dataset	NWPU VHR-10	Northwestern Polytechnical University Very High Resolution 10-Class Dataset
CDUPatch	Color-Driven Universal Adversarial Patch	ObjectSeeker	Certified Object Detection Defense
CornerNet	Keypoint-Based Anchor-Free Object Detector	PAD	Patch Adversarial Detection (defense)
CNCA	Customisable Naturalistic Camouflage Attack	PatchGen	Patch Generation Attack
CNN	Convolutional Neural Network	PatchGuard	Certified Patch Defense
CWA	Common Weakness Attack	PCA	Principal Component Analysis
DAC	Dual Adversarial Camouflage	R-CNN	Region-Based Convolutional Neural Network
DAP	Dynamic Adversarial Patch	RFLA	Reflected Light Adversarial Attack
DAS	Dual Attention Suppression	RGB	Red Green Blue (Visible Spectrum Imagery)
DB	Database	RP2	Robust Physical Perturbations
DE	Differential Evolution	RSI	Remote Sensing Image (Multi Scale Patch)
DE-DAC	Differential Evolution based Dual Adversarial Camouflage	RSOD	Remote Sensing Object Detection Dataset
DOTA	Dataset for Object deTection in Aerial images	SAC	Segment and Complete (Defense)
DPatch	Detector-targeting Adversarial Patch	SAR	Synthetic Aperture Radar
DTA	Differentiable Transformation Attack	SC-PCA	Shape and color Principal Component Camouflage Attack
ECAP	Environmentally Consistent Adversarial Patch	SGD	Stochastic Gradient Descent
EMA	Environmental Matching Attack	SSD	Single-Shot Multibox Detector
EOT	Expectation Over Transformation	Swin	Shifted Window Transformer
EVD4UAV	Evade Vehicle Detection in UAV Benchmark	TV	Total Variation
FCA	Full-coverage Camouflage Attack	UCDNet	Multi-UAV 3D Detection Network
FCOS	Fully Convolutional One-Stage Object Detector	UPC	Universal Physical Camouflage
HIT–UAV	High-Altitude Infrared Thermal UAV dataset	URAdv	Universal Robust UAV Adversarial Patch
IR	Infrared	VHR	Very High Resolution
IoU	Intersection Over Union	VisDrone	Vision Meets Drones Benchmark
		WB	White Box (attack setting)
NMS	Non-Maximum Suppression	V2X	Vehicle-to-Everything
